# Effects of Prevention Messages for Electronic Gambling Machines on Behaviors and Cognitions: Protocol for a Two-Arm Stratified Block: Randomized Controlled Study

**DOI:** 10.2196/75068

**Published:** 2025-11-10

**Authors:** Benjamin Galipeau, Christian Jacques, Serge Sévigny, Isabelle Giroux

**Affiliations:** 1 École de psychologie, Faculté des sciences sociales, Université Laval, Pavillon Félix-Antoine-Savard, local 1332, 2325 Allée des Bibliothèques, Québec, QC, G1V 0A6, Canada, 1-418-656-2131 ext 403251; 2 Centre québécois d'excellence pour la prévention et le traitement du jeu (CQEPTJ), Université Laval, Québec, QC, Canada; 3 Groupe de recherche en intervention et fondements en jeu (GRIF-jeu), Université Laval, Québec, QC, Canada; 4 Département des fondements et pratiques en éducation, Faculté des sciences de l'éducation, Université Laval, Québec, QC, Canada

**Keywords:** gambling prevention, responsible gambling, harmful gambling, pop-up messages, EGM, electronic gambling machine, primary prevention, preventive communication

## Abstract

**Background:**

Electronic gambling machines and online gambling are the reputedly most damaging gambling type from a public health perspective. Pop-up messages are often used as a responsible gambling (RG) measure to prevent harm for these screen-based types of gambling. Despite some evidence of effectiveness in the literature for these messages, limitations persist, among which low ecological validity is of particular concern.

**Objective:**

This study aims to test (1) the potential of pop-up messages as a prevention measure in a gambling setting and (2) whether this potential is moderated by characteristics of people exposed to the messages. Secondary objectives also tackle some fundamental assumptions of gambling studies conducted in a laboratory setting.

**Methods:**

This is a 2-arm stratified block randomized controlled study. In total, 80 participants are recruited under the false pretense of evaluating the realism of a gambling session in a laboratory replicating a bar. Duplicity is also used to make participants believe that they are risking their own money during the experimentation (ie, winnings and losses are real). Participants are randomized to one of the two arms in a 1:1 ratio: (1) experimental group (regular gambling session with prevention pop-up messages presented on a fixed schedule) and (2) active control group (regular gambling session). Outcomes measures include behaviors and cognitive and emotional responses to the pop-up messages. The believability of the gambling session’s realism is also evaluated.

**Results:**

Recruitment began in February 2024 and concluded in December 2024. Results are expected to be published in 2026. No results are currently available.

**Conclusions:**

This study will provide new insights on the efficacy of pop-up messages as a prevention measure for gambling as well as the appropriateness of laboratory studies as a substitute to a real-life setting.

## Introduction

### Background and Rationale

In the Diagnostic and Statistical Manual of Mental Disorders (DSM), gambling disorder refers to a persistent and recurrent problematic gambling behavior that disrupts several spheres of an individual’s life [1]. Whereas the dichotomous approach of the DSM may be useful in a clinical setting, it can easily lead to problematic subclinical behaviors not being considered. From a prevention standpoint, a continuous conceptualization of problem gambling is more useful. While cutoffs and nomenclature vary between existing screening tools, they mostly overlap with “Recreational” or “Non-problem” on the lower bound and “Pathological” reserved for severe problem gambling meeting the clinical threshold [2-8]. In between are the designations “At risk” and “Problem gambling”. The former regroups people experiencing some adverse consequences from their gambling habits and exhibiting some signs of impaired control. The latter designates people experiencing significant harm from or impaired control over their gambling habits, but at a subclinical level [8]. Finally, recent developments in the literature have begun to shift the focus from counting people afflicted by them to counting harms (see the Conceptual Framework of Harmful Gambling for more information [9]). This framework allows for a more granular study of the impacts of gambling, notably the recognition that relatively small harms can be significant when experienced by a large population.

Data from a recent meta-analysis indicates that the worldwide prevalence in the adult population for any risk gambling (ie, anything higher than recreative or no problem gambling) is 8.7% and is 1.41% for problem gambling [10,11]. Problem gambling is associated with a range of negative effects encompassing multiple domains. For example, Cowlishaw and Kessler [12] found associations between problem gambling and poorer outcomes on mental health, psychosocial, and financial indicators. These effects are largely confirmed in a recent systematic review by Allami et al [13]. More generally, recent frameworks of gambling harms have been developed to classify and itemize different gambling harms across dimensions and temporal spans [14], among which the taxonomy of harms by Langham et al [15] is considered especially thorough. These frameworks of gambling harms are especially useful to show that harms are not limited to the individual gambler but can also affect their immediate surroundings and society at large. Furthermore, harms are not limited to pathological gamblers and can persist even after complete abstinence from gambling. To limit and prevent these harms, a wide range of measures grouped under the term “responsible gambling” (RG) have been put in place (see the study by Blaszczynski et al [16] for a detailed definition of the RG concept and see the study by Williams et al [17] for a review of different RG measures).

Among RG measures are pop-up messages. In the context of RG, pop-ups, dynamic warnings, or on-screen messages are presented during gambling activities to prevent or reduce gambling-related harm [18-20]. A common strategy used when designing such messages is providing factually accurate information used to correct cognitive distortions and irrational beliefs about gambling (eg, “The way you play will not increase your chances of winning” [21]). Other pop-up messages prompt gamblers to set limits on time or money allocated to gambling and then reappear when those limits are reached (eg, “You have reached your preset limit of 20 credits. You still have 60 credits in the slot machine…” [22]). Pop-up messages have also been used to remind gamblers about their behavior in-play (“You have now played 1000 slot games. Do you want to continue?” [23]) with the rationale that this combats compulsive play and dissociative-like state [24-27]. This strategy is sometimes coupled with normative feedback (eg, “…Only a few people play more than 1000 slot games” [28]). Finally, pop-up messages have used self-appraisal wording to foster self-awareness in gamblers by inducing a deeper and more personal processing of the information (eg, “Have you spent more than you can afford?” [29]).

The effectiveness of pop-up messages is still debated in the literature. On one hand, some results suggest that pop-up messages can be an effective and minimally invasive strategy for introducing responsible gambling information and reinforcing responsible gambling behaviors [18-20]. They also have the potential to reach a wide variety of gamblers at minimal cost and can be tailored to better fit their receiver’s characteristics (eg, age, sex, etc) and the context in which they are presented (eg, if the message is presented during a winning or a losing streak). Furthermore, the interest in pop-up messages is heightened by the fact that they serve as a prevention strategy for types of gambling regarded as the most damaging from a public health perspective, namely electronic gambling machines (EGMs) and online gambling [10,30-33]. On the other hand, numerous methodological limits affect the RG pop-up messages literature, including the paucity of replication studies for a same message parametrization [18-20], use of self-reported data instead of objective observations (eg, [29,34]), limited sample sizes (eg, [35]) and ecological validity flaws (eg, [36-38]). Thus, it is evident that more data is necessary to evaluate the effectiveness of RG pop-up messages and their potential moderators.

### Designing Effective Prevention Pop-Up Messages

While the literature is not settled on the best design for effective RG pop-up messages, some elements are more supported than others. To synthesize available data in a useful manner, it is pertinent to start with an overarching model. For this paper, we based our strategy on Comprehensive Messaging Strategy For Sustained Behavior Change (CCSSBC) [39], a multitheoretical persuasive communication strategy combining both message tailoring strategy of the Transtheoretical Model (TTM) [40] and message framing strategy of the Self-Determination Theory (SDT) [41,42]. “Tailoring” is the adaptation of messages’ content to better fit the characteristics (eg, sociodemographics, psychological, etc) of its target individuals or populations [39]. On the other hand, “framing” refers to the way a message is conveyed, not its content per se [39].

The TTM describes the process of change in human behavior and proposes tailoring interventions to the stage of change of the receiver [40]. In their review, Pope et al [39] build on this model and propose 4 stages: detection (characterized by unawareness of a problem or failure, willfully or not, to acknowledge said problem); decision (characterized by acknowledging a problem exists, which leads to a state of uneasiness, and therefore higher receptiveness to information on potential solutions or reliefs); implementation (where the intentions about the problem are formed but have still not been enacted); and maintenance (where behavior is produced and the challenge is now about sustaining in the long term). Tailoring RG messages to gamblers’ stage of change would require some form of evaluation that would also need to be periodically updated. This could be done by a questionnaire via a gambling website or tied to an identity card required to play EGMs (eg, [43,44] for the use of a loyalty card to track behavior on EGMs). While this kind of evaluation would be desirable, it is not actually done by most, if any, gambling website, even less for offline play. Therefore, the next best option would be to tailor messages to the stage of change of gamblers that would benefit the most from it: gamblers at the detection stage. As said earlier, RG pop-up messages are a low-level prevention tool pushed to gamblers while in play, without any action needed on their part to receive them. This makes RG pop-up messages ideal for those who are the most oblivious to their own gambling behavior and are the least inclined to search for protective strategies by themselves. Gamblers at other stages of change could still benefit from these messages even though they are less tailored to them. This (presumably) reduced effectiveness is thought to be offset by the increased awareness of gamblers at higher stages of change, which in turn should make them more autonomous in protecting themselves and less reliant on the “imposed” protection of RG pop-up messages.

SDT describes how people regulate and act according to their social environment, goals, and motives [41]. Broadly speaking, both goals (what behavior is done) and motives (why said behavior is done) can be intrinsic (comes from the self, for their own sake) or extrinsic (determined by outside influences, like shame from peers). In their review, Pope et al [39] show that messages fostering both intrinsic goals and motives lead to better outcomes by almost every metric (ie, increased well-being and quality of life, less stress and anxiety, deeper processing of information, more commitment to positive change, and sustained positive behavior, etc). Whatever stage of change, messages should always strive to foster intrinsic-oriented goals and motives. This is readily compatible with the inclusion of self-appraisal statements in RG messages, which are the most promising feature in the literature for this specific prevention tool [28,29,45-47]. At the detection phase, Pope et al [39] suggest that messages should “(a) identify intrinsic risks or negative consequences associated with avoiding the behavior; (b) introduce small feasible options that serve as solutions to the problem; (c) provide a self-determined rationale; and finally, (d) use images/titles that reflect intrinsic goals, such as fitness and well-being, as opposed to appearance, social comparisons, or appealing to others.”

Apart from the “content” portion of effective RG pop-up messages are the “container” features. These features refer to strategies aimed at better capturing the attention and maximizing readability and easiness of understanding. First is the necessity to periodically disrupt the game by forcing a pause. An explication for why EGMs are so much linked to problem gambling is their capacity to induce dissociative-like state, which makes gamblers lose track of time and money spent [24-27]. Therefore, a good pop-up message should be disruptive enough to break this mental state and give enough time to gamblers to think more rationally about their behavior. This is exemplified by Williams et al [17], who argue that smoking bans in public spaces could have been one of the more effective RG measures, because it forces gamblers, who are predominantly smokers, to take pauses outside gambling venues. Having a minimum duration between the appearance of the RG pop-up message and being allowed to continue is a common feature in the literature. Sometimes, the forced pause was as effective as the pause plus RG message condition to reduce gambling behavior [21,48]. The duration of the pop-up message should be sufficient, so gamblers have enough time to read and reflect on them, but still not be so long that they become excessively obstructing. Similarly, presentation frequency should be sufficient so that gamblers are exposed to them but not so much as they become harassing. There is much variation on these two parameters in the literature. On the higher end are studies like Ladouceur and Sevigny [48] with a 7s message each 15 games and on the lower end are studies, like Auer et al [23] or Schellink and Schrans [49], with a single message after a continuous hour of play and no minimum duration. A common observation by researchers was that few participants were exposed to their RG pop-up messages (eg, [23,28,49]). Schrans et al [50] tried a 30-minute presentation frequency that had the benefit of elevating the proportion of gamblers exposed to the RG pop-up messages but failed at lowering gambling behavior more than the 1-hour presentation frequency.

RG pop-up placement should be such that they are readily noticeable (other than the fact that they block the gambling activity). Gainsbury et al [34] compared the performance of RG pop-up messages presented in the center versus on the periphery of EGMs’ screens. Pop-up messages at the center of the screen were more noticeable and remembered by participants and were perceived as more useful. Participants exposed to the centered pop-up messages also reported gambling less than those exposed to the peripheral ones.

The combination of a signal word [51] and a pictogram [51-53] can be useful to capture attention. Data on the subject mainly comes from the products safety literature where physical injuries are the main concerns. Because gambling deals with psychological and social harms which can nevertheless be devastating, the lower-level signal word “Notice” (prescribed by ANSI for practices not related to physical injury [54]) could be too tame to convey the proper level of danger. We argue that higher-level signal word “Caution” (prescribed by ANSI for hazardous situations which, if not avoided, could result in minor or moderate injury [54]) is more adequate for this purpose.

To improve readability, messages should be designed with an appropriate text-background contrast, font, font size, letter case, and text disposition [51,55]. Because people’s perceptual abilities can vary, it is preferable to design messages to maximize the number of people that can read them easily. Messages accessible for disadvantaged people can still be easily read by the regular population, while the reverse is not necessarily true [56]. Good text-background makes the message stand out [51,55] and improves information processing [57]. Black characters on a white background are considered easier to read than white characters on a black background [57].

There is no consensus in the literature regarding which of serif or sans-serif fonts are optimal for readability [58-61]. However, a more recent study by Rello and Baeza-Yates [62] found that sans-serif fonts were easier to read for people with dyslexia. Current ANSI norms also recommend sans-serif fonts [54].

Font size should be optimized so that under normal reading conditions the message is entirely contained in one’s field of vision [60,61]. Because readability tends to increase with font size [60,61,63], selected font size should err on the higher side of the recommended size [54].

Messages should be written with a mix of capital and lower-case letters. The general rule is that lower-case letters are more distinguishable from one another than capital letters [55]. Capital letters are more useful to mark the beginning of phrases or statements in the messages, or to emphasize the signal word in the message’s header [54].

Wogalter et al [51] recommend segmenting messages by units of meaning written in a simpler telegraphic style and disposed of in a point-form manner. Text should be left-aligned. This disposition allows for an easier identification of each “argument” of the message and improves memorization. The ANSI proposes using bullet points to highlight even more each message segments [54].

Finally, designing effective messages necessitates accounting for habituation. Indeed, repeated exposure to prevention messages has the potential to lower their effectiveness because people pay less attention to them [64]. Hitchman et al. [64] suggest that an optimal strategy is to circulate different messages at the same time and periodically change the whole set of messages for a new one.

### Ecological Validity

Ecological validity poses a more complex problem than parametrization of the “ideal” prevention pop-up message. Testing the effectiveness of RG pop-up messages in a natural environment poses major ethical, organizational, and financial challenges, mainly related to the need to reach an agreement with a gambling operator. Testing in a natural environment makes inevitable some sort of collaboration with a gambling operator to access their infrastructure and player base. This places the researchers at the mercy of the gambling operator who can interfere with the prevention measure tested or the research design itself. For example, in their study testing the effect of RG messages on EGMs in real gambling venues, Gainsbury et al [29] were asked by one of the venues’ owners to reduce the presentation from 15 seconds four times per hour to 10 seconds once per hour. Even without such interference, these sorts of arrangements can incentivize researchers to self-censor to maintain access to their partnered gambling operator’ infrastructure for future studies [65,66]. Finally, there are technical difficulties regarding gambling operators’ proprietary software that can limit the possibilities of alterations to insert RG pop-up messages. For the aforementioned reasons, it is generally more practical to test RG measures in a laboratory setting, even if this approach comes with its own set of problems related to ecological validity. Indeed, recent systematic reviews evaluating the effect of RG pop-up messages show that most studies have been conducted in a laboratory environment, whereas only a few were conducted in a natural setting [18,19].

In laboratory settings, volunteers are typically invited to gamble by wagering virtual credits (eg, [47,67]) or their monetary compensation received for their participation in the study (eg, [21,35,36,48,68]). Their behavior in that setting is then deemed to resemble that of gamblers in a real gambling environment. Surprisingly, this proposition has received very little scientific verification. Data suggests that gamblers would be more emotionally engaged in play in a real gambling environment [69,70] but would take more risks in a laboratory setting [71]. These results could be explained in part by the fact that laboratory research in the field of gambling neglects a fundamental aspect of its definition: the wagering of money or an object of value, a wager which, once placed, cannot be taken back [72]. Indeed, a constant of laboratory studies is both the absence of monetary risk and low possible prizes. In the best of cases, wagering a certain amount of money provided upfront only risks participants losing something that was not in their possession before participating in the study. This is, by definition, enforcing some kind of preset limits as it is not possible for participants to bet money they cannot afford to lose. In cases where only virtual credits are gambled, the disconnect with risk is even greater. Prize money, for its part, is never really addressed in laboratory studies. Maximum winnable prizes are often quite low (eg, AU $40 [47], some chances to win a 19-inch TV [36], etc) compared to what is theoretically possible in a real gambling environment (eg, in Quebec, Canada, maximum jackpot per game on video lottery terminals, a subtype of EGMs, is CA $1000 [73]).

The absence or reduction of participants’ sense of risk while gambling in a laboratory setting could reduce people’s gambling inhibitions. This raises doubts about the seriousness with which they would take any warning message about the consequences of their actions. Indeed, without any serious motivation to gamble (eg, desire to win big, desire to make up for a major loss, etc), it is quite possible that participants would be more intensely subject to what Orne [74] calls “demand characteristics of the experimental situation,” which are “…the totality of cues which convey an experimental hypothesis to the [participant and] become significant determinants of [participants’] behavior...” Participants exposed to RG measures in a laboratory setting could understand that they are being encouraged to moderate their gambling by the researchers. Participants could then modify their behavior mainly to either confirm or contradict the researchers’ hypotheses. This bias could artificially inflate the effectiveness of certain RG measures when tested in the laboratory. These RG measures may subsequently fail to fulfill their protective role when deployed in the field.

Apart from the sense of risk, most laboratory studies on RG prevention messages were conducted in a significantly lesser immersive or restrictive environment. For example, in the study by McGivern et al [38], participants were proposed a computerized gambling task. In the study by Cloutier et al [21] and Ladouceur and Sevigny [48], participants gambled on real EGMs in a properly decorated gambling environment, but the gambling session was limited to how long it took to respectively spend CA $20 and CA $10. At this point, it is worth noting that Bjørseth et al [18] did not find a significant moderation effect of setting (real gambling setting vs laboratory). However, this absence of effect could as easily be explained by the extreme unexplained heterogeneity between studies. More studies are needed to determine the source of this heterogeneity.

This study addresses the aforementioned ecological validity concerns in multiple ways (see “Duplicity” and “Gambling session in the laboratory” subsections of the “Methods” section for details). First, the study’ environment replicates as closely as possible a real gambling setting. This is done by using real EGMs and giving attention to decor and ambiance (eg, lighting, music, etc). Second, participants are allowed to behave in a more natural and unrestricted way. For example, they can take pauses from gambling without ending the study, they are not informed about the maximum duration that the protocol allows for gambling, and they are told they can gamble as much as they want. Finally, and most importantly, efforts are done to both elevate the perception of personal risk and provide a comparable incentive to gamble as a real gambling setting. The elevation of risk is done by use of duplicity to make participants believe they are betting their own money. Participants are also provided with a way to withdraw more money from their bank account to prevent situations where they would end their gambling session, not because they chose to, but because they did not bring enough money with them. An incentive to gamble is done by offering a comparable jackpot to the one offered in local gambling venues.

### Aims and Objectives

This study aims to test (1) the potential of pop-up messages as a prevention measure in a gambling setting and (2) whether this potential is moderated by characteristics of people exposed to the messages. Secondary aims also tackle some fundamental assumptions of gambling studies conducted in a laboratory setting.

Specifically, the main objectives are to test to what extent prevention pop-up messages affect participants:

1. Gambling behaviors (eg, money betted, gambling session length, gambling intensity, etc).
2. Cognitions (eg, thoughts elicited by messages, perceived effectiveness of message, etc).
3. Emotions (eg, enjoyment of gambling, emotional response to messages, etc).

Secondary objectives are:

1. To test to what extent the main effects are moderated by participants’ characteristics (eg, gender, age, education, Problem Gambling Severity Index [PGSI] category, etc) moderate the main effects found in objectives 1‐3.
2. To examine the feasibility of studies conducted in a laboratory setting with the use of real money (or using deception to make the participants believe they are gambling their own money). This objective is accomplished with the use of five research questions:
  - 5.1. Is it possible to recruit enough participants for the study to conduct meaningful statistical analysis (ie, does the novel approach of risking one’s own money in a study without guaranteed compensation deter too many people from participating)?
  - 5.2. Does the laboratory bar replicate adequately the overall “vibe” of a typical gambling venue with EGMs?
  - 5.3. Do the participants find their actual gambling activity in the bar replica as realistic as a real one?
  - 5.4. Do the participants believe they are gambling their own money during the study (ie, to what extent does the deception work)?
  - 5.5. Do outcomes, both self-reported (past 12 months) and measured in the laboratory, differ from each other?

## Methods

### Ethical Considerations

This study was approved by the Comités d'éthique de la recherche avec des êtres humains de l'Université Laval (CÉRUL; reference 2020-076 A-1 / 27-05-2024). Preliminary consent is obtained verbally during the intake interview and confirmed with a signature just before the gambling session in the laboratory. Some aspects of the study are withheld from the participants and are only revealed during debriefing after the gambling session has ended (see "Duplicity" and "Gambling session in the laboratory" subsections of the "Methods" section for details). After debriefing, participants are asked to confirm their consent one last time. Participants can remove consent at any point of the study without prejudice. After debriefing, participants who wish to quit without completing the study can ask that all their data be destroyed. Compensation for participation can vary between CA $30 and CA $500 depending on the results of the gambling session, which is completely random (see "Presentation of the Study and Verbal Consent" and "Duplicity" subsections of the "Methods" section for details). Some transportation expenses were also reimbursed on top of the compensation, namely transit fare, parking validation at the university, and taxi for the elderlies and people with mobility impairments. All data is kept in secured servers (files) and locked drawers (physical forms). Participants' data is anonymized with a nonsequential ID number.

This study exposes participants to potentially stressful situations. Notably, by gambling with their own money, participants risk gambling more than what they can afford and find themselves in a situation where they think they have lost a lot of money. This stress is a byproduct of the duplicity required to ensure maximal ecological validity of the study. It is mitigated by the fact that any potential stress is limited to a maximum of 2 h (the time limit for the study), after which debriefing ensues and they learn their “lost money” will be returned to them.

The use of duplicity poses some concerns over the importance of free and informed consent. While participants initially enroll in the study without knowing all the parameters, they have the option to retroactively remove their consent and ask for the destruction of their data during the debriefing. Some steps are also taken to minimize aspects of the study that are masked by duplicity. For example, even though losses are reimbursed to participants, gains are still paid as advertised. This prevents a potential situation where a participant who wins a lot of money and expects to leave with it is instead given a standardized (and lesser) amount.

It is also worth noting that this study does not expose participants to higher levels of risk and stress than what they already frequently experience in their own lives. Inclusion criteria target participants who regularly gamble on EGMs in their everyday lives. In the context of the study, participants are provided with the same incentives to gamble, but nothing is done to exacerbate these behaviors (eg, altering the EGMs’ payout rate, offering loans to gamble, etc). On the contrary, half the participants are provided with prevention messages, and any money lost while gambling is reimbursed at the end of the session. Also, because this study is conducted in a psychology laboratory, people on site can provide crisis support should a participant feel unwell after a significant loss. This kind of support is not usually available where EGMs are situated. Finally, we chose not to recruit the most vulnerable gamblers (ie, people trying to reach or maintain abstinence). Despite those protections, the ethics committee felt that probable pathological gamblers should also be excluded from the study.

### Study Design

This is a 2-arm stratified block randomized controlled study. Participants are randomized to one of the two arms in a 1:1 ratio:

- Experimental group: Regular gambling session with prevention pop-up messages presented on a fixed schedule.
- Active control group: Regular gambling session.

**Figure 1.**
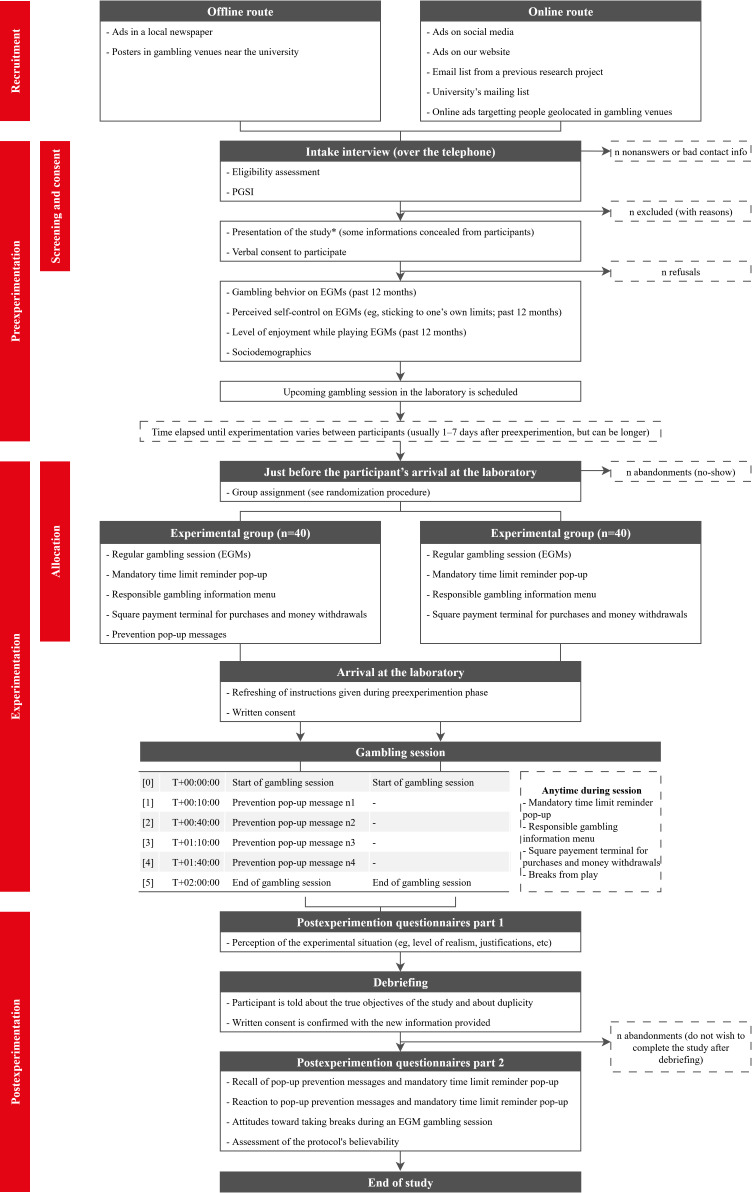
Schematic overview of the 2-arm stratified block randomized controlled trial. EGM: electronic gambling machine; PGSI: Problem Gambling Severity Index.

There are 3 assessment phases in this study (see Figure 1). Recruitment began in February 2024 and concluded in December 2024. Study completion is expected in February 2026. Study outcomes will be reported in line with the Consolidated Standards of Reporting Trials guidelines and the extension for social and psychological interventions trials (CONSORT-SPI) [75].

### Sample Size Considerations

Using G*Power, we estimated the required sample size for an α level of 5% and ß level of 80% for a 2-tailed *t* test for a range of effect sizes (see Table 1). This was done both for testing differences in outcomes between 2 independent groups and for repeated measures.

**Table 1.** Required sample size for a range of effect sizes.

Effect size (*g*)^a^	Per group, n (%)	Total, N
0.2	394 (50)	788
0.3	176 (50)	352
0.4	100 (50)	200
0.5	64 (50)	128
0.6	45 (50)	90
0.7	34 (50)	68
0.8	26 (50)	52
0.9	16 (50)	32

a Calculated for an α level of 5% and ß level of 80% for a 2-tailed *t* test.

A recent meta-analysis by Bjørseth et al [18] on the effects of RG pop-up messages on gambling behaviors and cognitions reports effect sizes respectively of *g*=0.62 (95% CI 0.36-0.87 and *g* 0.58 (95% CI 0.33‐0.83) when adjusting for publication bias. Based on those values, the total sample size would be between 86 and 96. Note that sample size requirements for 1-way analysis of covariances (ANCOVAs) and repeated measures analyses would be equal to or smaller. Considering the potential difficulty in recruiting participants accepting to play with their own money, we aimed at a sample size of 80. Therefore, we can expect a power range from 72.34% to 77.66% with effect sizes comparable to what is reported by Bjørseth et al [18].

### Participants and Eligibility Criteria

We aim to recruit 80 participants in total. While many may refuse to participate when presented with the study’s specifics, we do not anticipate a significant number of dropouts after consent is obtained. Textbox 1 shows the inclusion and exclusion criteria.

Textbox 1. **Eligibility criteria.**

Inclusion criteria:

- Aged 18 years and older.
- Functional literacy in French.
- Having played electronic gambling machines (EGMs) at least once every 2 weeks for the past 12 months. Note that this criterion was subsequently lowered to having played EGMs at least 6 times in the past 12 months to expand the recruiting pool. This change was done on May 27, 2024.

Exclusion criteria:

- Classified as a probable pathological gambler (score ≥8 on the Problem Gambling Severity Index, PGSI).
- Currently receiving treatment for problem gambling or trying to achieve or maintain abstinence from gambling.
- Currently under self-exclusion from a gambling venue or website.

The rationale for the age cut-off is that it is the legal age to gamble where the study is conducted (Quebec, Canada). The literacy requirement is justified by the fact that this study is conducted in French and requires being able to read and understand simple texts in that language. Questions are also asked and answered in French.

The age cut-off is the legal age to gamble where the study is conducted (Quebec, Canada).

The rationale for a floor limit on play frequency is that prevention pop-up messages are expected to produce a small effect that becomes more impactful when cumulated over multiple sessions. Therefore, these messages are thought to be more suited to people who gamble on a regular basis. Furthermore, regular EGM gamblers are more likely to feel compelled by a message warning about the dangers of excessive gambling than people who gamble very sporadically.

The rationale for a floor limit on play frequency is that prevention pop-up messages are expected to produce a small effect that becomes more impactful when cumulated over multiple sessions. Therefore, these messages are thought to be more suited to people who gamble on a regular basis. Furthermore, regular EGM gamblers are more likely to feel compelled by a message warning about the dangers of excessive gambling than people who gamble very sporadically.

The rationale for the exclusion criteria is ethical considerations. The first criterion is mandated by the ethics committee to prevent possible adverse effects on the most vulnerable gamblers. The other 2 criteria aim not to interfere with someone’s recovery process or wishes to stop gambling.

### Recruitment

From February 2024 and on, the study was advertised online via social media, our research team’s website, and our university’s mailing list. An email was also sent to participants from a previous study who agreed to be contacted again. The study was advertised offline via ads in a local newspaper 2 times in February 2024. Recruitment posters were placed in gambling venues in the same city of our university.

To boost recruitment, we started a second recruitment wave during the summer of 2024. This wave mainly used online ads targeted at people geolocated at gambling venues. Inclusion criteria were also relaxed on minimal gambling frequency (see the “Participants and Eligibility Criteria” section).

### Preexperimentation Phase

#### Screening

Screening and following interview for the preexperimentation phase are done over the phone. Participants are asked questions to determine if they satisfy the inclusion and exclusion criteria. Not all criteria are communicated on the recruitment ads, and neither are the participants told beforehand what answer would disqualify them. The screening interview ends with the PGSI, which is a subscale of the Canadian Problem Gambling Index [6]. This instrument is designed to determine problem gambling severity and is the actual gold standard for problem gambling screening in the general population. The instrument is composed of nine items scored on a 5-point Likert scale. Scores range from 0‐27 and classify gamblers in four categories: (1) nonproblem (score of 0), (2) low risk (score of 1‐2), (3) moderate risk (score of 3‐7), and (4) problem gambling (score ≥8).

#### Presentation of the Study and Verbal Consent

After satisfying the screening criteria, participants are presented with the details of the study. Participants are recruited for a single gambling session in a bar replica equipped with EGMs. Recruitment is done under the false pretense of evaluating the realism of a gambling session in a laboratory replicating a bar. The main points communicated to the participants during the presentation of the study are:

- Gambling is done with your own money. You must bring the amount they wish to gamble the day of the experiment.
- Winnings are paid up to CA $500 more than the total amount inserted in the EGM over the course of the gambling session (eg, if you insert CA $60 in the EGM during your session, you could be paid up to CA $560). In turn, losses are real. There is no baseline compensation for participating in the study. The only compensation for participation is the potential winnings made while gambling. Overall, it is possible to end the study with less money than at the beginning, even to lose it all.
- The EGMs used in this study are of the same model currently in operation in Quebec, Canada. Their payout rate is the same (92%).
- You are free to gamble as much and for as long as you like.
- You are allowed to take breaks.
- Snacks and nonalcoholic beverages can be purchased with real money during the study via cash or electronic transactions.

Participants who give their verbal consent proceed with the intake interview.

#### Duplicity

In total, 4 aspects of this study are hidden from participants to emulate a realistic level of personal risk and prevent demand characteristics.

First, the main objectives of this study, namely “Evaluating the effect of prevention pop-up messages on behaviors and cognitions,” are hidden from the participants. Instead, they are told that this study is about evaluating the bar replica (ie, the ambiance, look, functionality, etc) and the gambling session’s realism. They are also told that this information will be used to inform future studies using the same bar replica. To prevent participants from mistaking the prevention pop-up messages for a bug or becoming skeptical about the study’s true objectives, they are told that some characteristics of the EGMs could differ slightly from their typical experience. It is also specified that these differences are not guaranteed to be present (to account for participants in the control group). The explanation given to participants for potential differences is that our research team is conducting multiple parallel studies with the same EGMs and that it would not be practical to tinker with the EGMs between each participant. These potential differences are kept vague and presented to the participants as minor modifications, mostly affecting the user’s interface (eg, change in fonts to improve readability) and never affecting the games’ mechanics and payout rates.

Second, participants cannot lose their own money. All money inserted in the EGMs during the gambling session is given back at the end of the study, though winnings are paid as advertised (see Table 2). Also, participants who incur net losses or winnings less than CA $30 are reimbursed their money and given exactly CA $30. This ensures a floor limit on the compensation for participating in the study.

**Table 2.** Examples of payments made to participants under different scenarios.

Amount inserted in the EGM^a^ by the participant during the gambling session (CA $)	Amount left on the EGM at the end of the gambling session (CA $)	Net gains^b^ (CA $)	Theoretical maximum amount payable to the participant^c^ (CA $)	“New” money gained by the participant^d^ (CA $)	Total amount given to participant at the end of the gambling session^e^ (CA $)
60	120	+60	560	60	120
60	800	+740	560	500	560
60	65	+5	560	30	90
60	0	–60	560	30	90

a EGM: electronic gambling machine.

b Money remaining minus what was inserted in the EGM during the gambling session.

c Payable net gains are capped at CA $500. This amount is therefore CA $500+ money inserted in the EGM by the participant during the gambling session.

d Money gained by the participant that they did not have before the gambling session.

e The amount inserted in the EGM is returned to the participant (CA $60 in all examples) + stipulated compensation.

Third, the gambling session’s duration is limited to 2 hours. The gambling session starts when the participant inserts money for the first time in the EGM. Breaks during the gambling session are permitted (eg, using the restroom, buying snacks from the bartender or barmaid, etc) but are included in the 2-hour time limit. If the participants have not yet ended their gambling session upon reaching the 2-hour time limit, they are offered to take a short break to answer some questions. They then proceed to the postexperiment phase. If the participant insists on continuing to play or tries to quit hastily (eg, because they are angry about losing money), they are prematurely debriefed before proceeding with the postexperiment phase.

Fourth, unbeknownst to them, participants are monitored through a secret camera hidden in the ceiling during their gambling session. Their gambling behavior is also recorded from the EGM they play on.

#### Intake Interview

The intake interview collects information on basic sociodemographic factors, gambling behaviors on EGMs (past 12 months), general level of fun while playing EGMs (past 12 months), and perceived self-control while playing EGMs. Participants then schedule a gambling session to be done in our laboratory on the university’s campus. While free to choose any date and time they wish, participants are encouraged to select a moment that fits their usual gambling habits. There is no maximum or minimum time limit between the intake interview and the gambling session in the laboratory. Sessions are usually scheduled days to weeks after the intake interview.

### Experimentation Phase

#### Randomization

**Figure 2.**
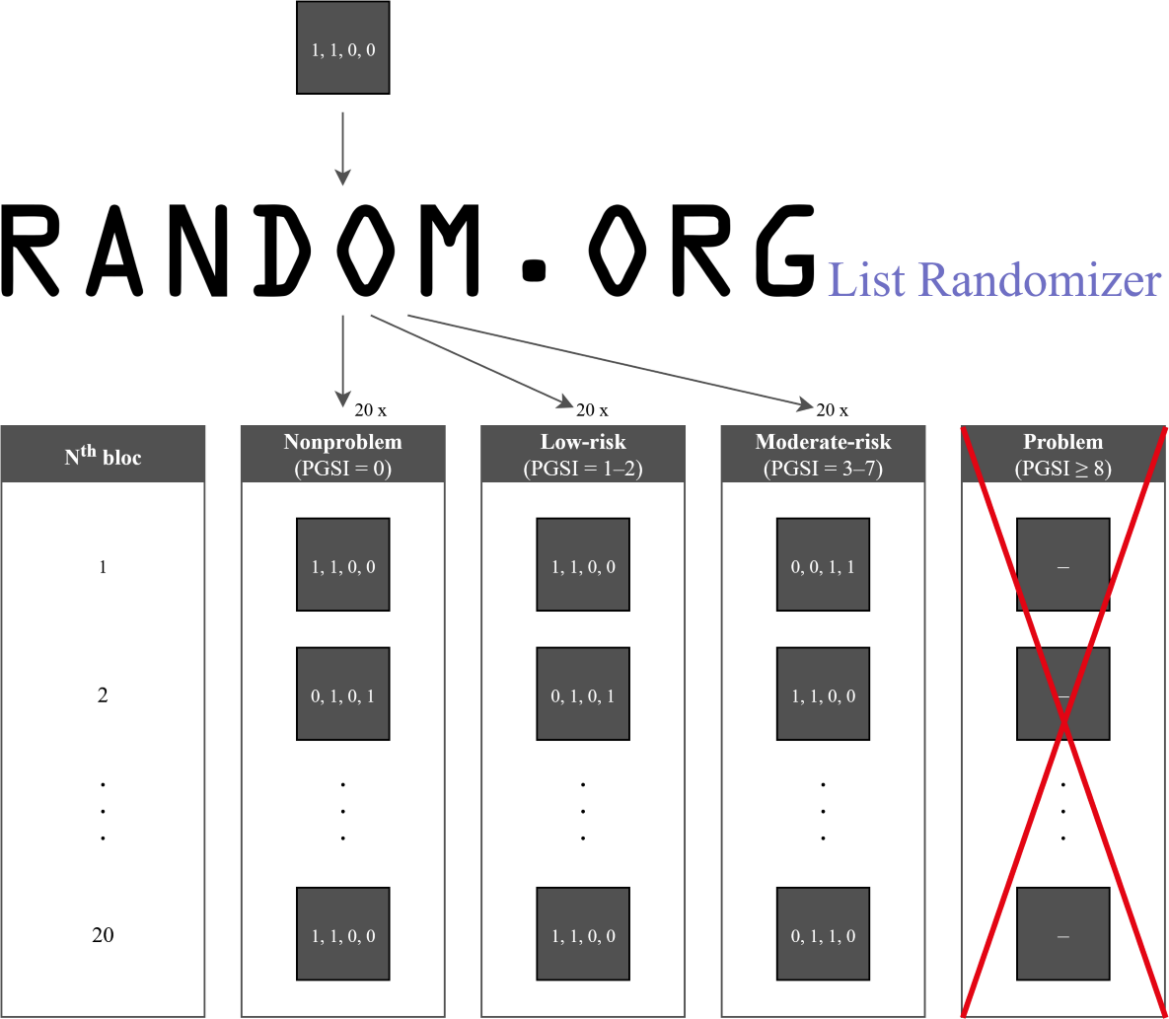
Randomization procedure. PGSI: Problem Gambling Severity Index.

Blocks are size 4 and strata are divided on problem gambling severity using PGSI categories. Randomization was done using RANDOM.ORG’s list randomizer tool. The list within each block, “1, 1, 0, 0” (where “1” is experimental condition and “0” is control), was randomized 20 times for each PGSI category, excluding the probable pathological gambling category (see Figure 2). This arrangement of 80 places for the three lowest PGSI categories allows for any combination of levels of problem gambling among recruited participants while preventing major imbalance between the 2 groups on this characteristic.

**Figure 3.**
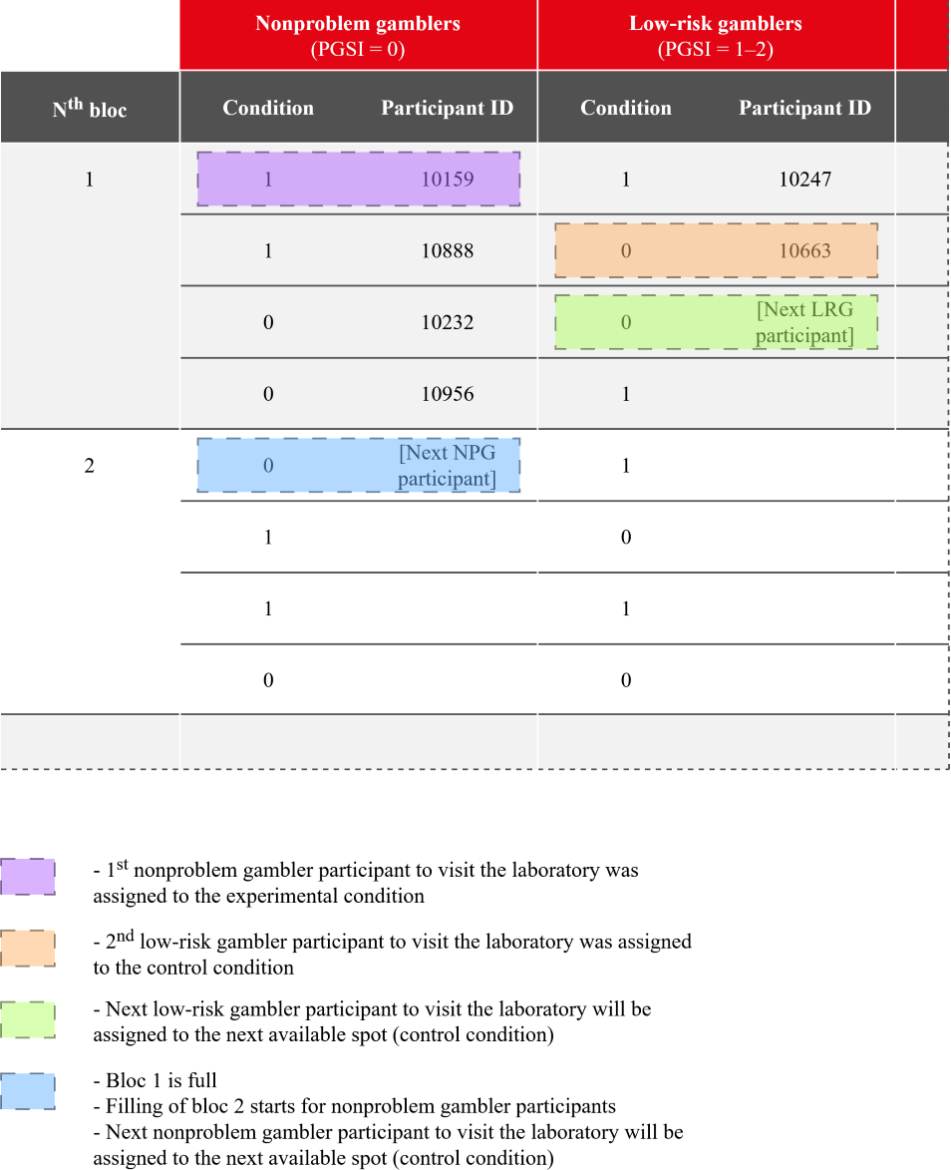
Allocation procedure. PGSI: Problem Gambling Severity Index; NPG: nonproblem gamer; LRG: low-risk gambler; Condition 1: Experimental; Condition 2: Control.

Allocation is done just before the participants arrive at the laboratory, on the day of the experiment. Participants are allocated to the first available spot from the top of the randomized list corresponding to their PGSI category (see Figure 3). Participants who complete the intake interview but do not show up at the scheduled appointment vacate their spot on the list. They may then be reassigned depending on whether they reschedule another gambling session. Meanwhile, the vacated spot becomes available for another participant who fits the same profile. The moment participants present themselves to the laboratory, their spot on the list cannot be reallocated, even if they desist later in the process.

#### Concealment of Allocation and Blinding

While participants in the experimental group should notice the novel prevention pop-up messages, for all they know before debriefing, there is no “assignment” per se. This is because the study is presented as an opinion survey about the realism of the bar replica and the gambling session in it. No instruction suggests any type of comparison between conditions. Therefore, participants would not be aware that there are 2 different groups, nor that this study tests the effects of prevention pop-up messages.

Note that masking becomes “open label” after debriefing. Indeed, after being informed of the true goals of this study, participants are easily able to determine in which group they were allocated based on what occurred during their gambling session. Regardless, gambling behaviors are recorded before debriefing and would not be affected by unmasking. There is no functional way to mask group assignment to the research team. However, the randomization procedure ensures that allocation is purely random.

#### Gambling Session in the Laboratory

**Figure 4.**
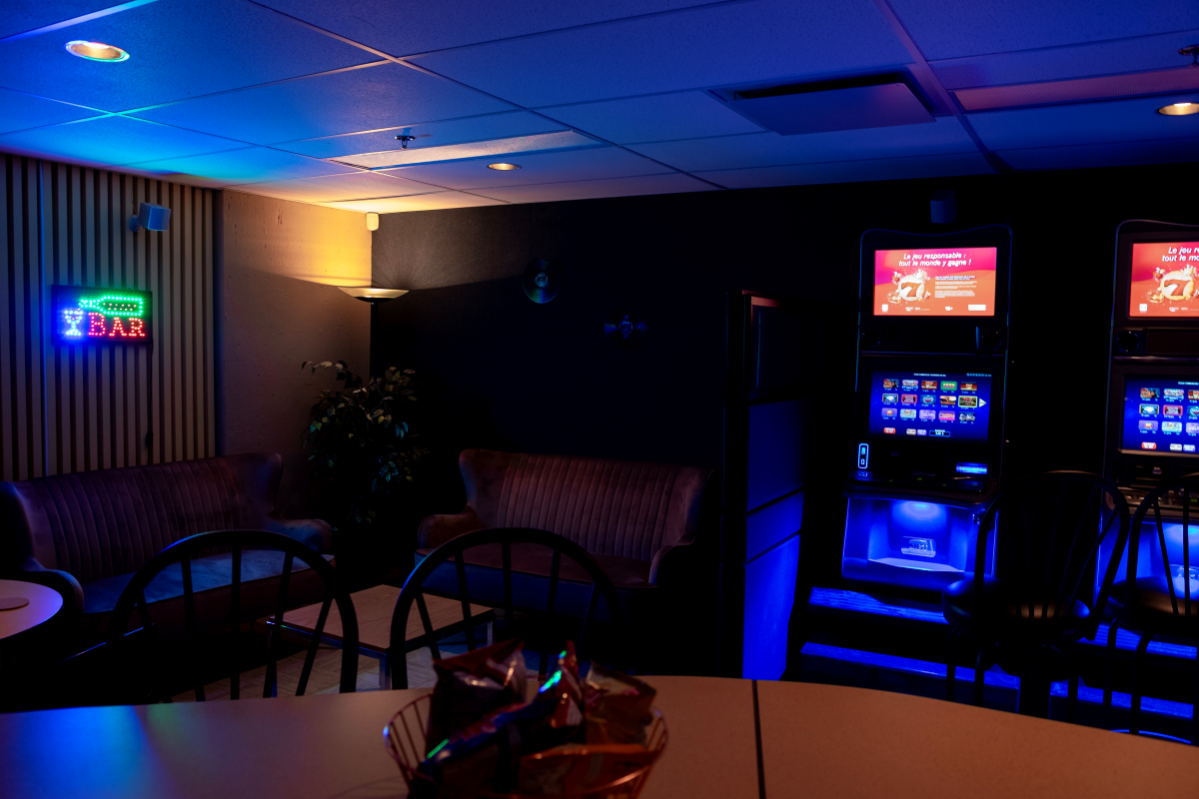
Bar environment—electronic gambling machines and lounge.

There is only a single participant at a time in the laboratory for the gambling session. Each participant arrives at the lab at a scheduled appointment. Then, a research assistant greets the participant and proceeds to refresh their memory about the important aspects of the study. The participant is then brought to a room replicating a typical bar with EGMs (eg, subdued light, music, bar paraphernalia, fake liquor on display, etc; see Figures4  5 and Multimedia Appendix 1 for additional images).

**Figure 5.**
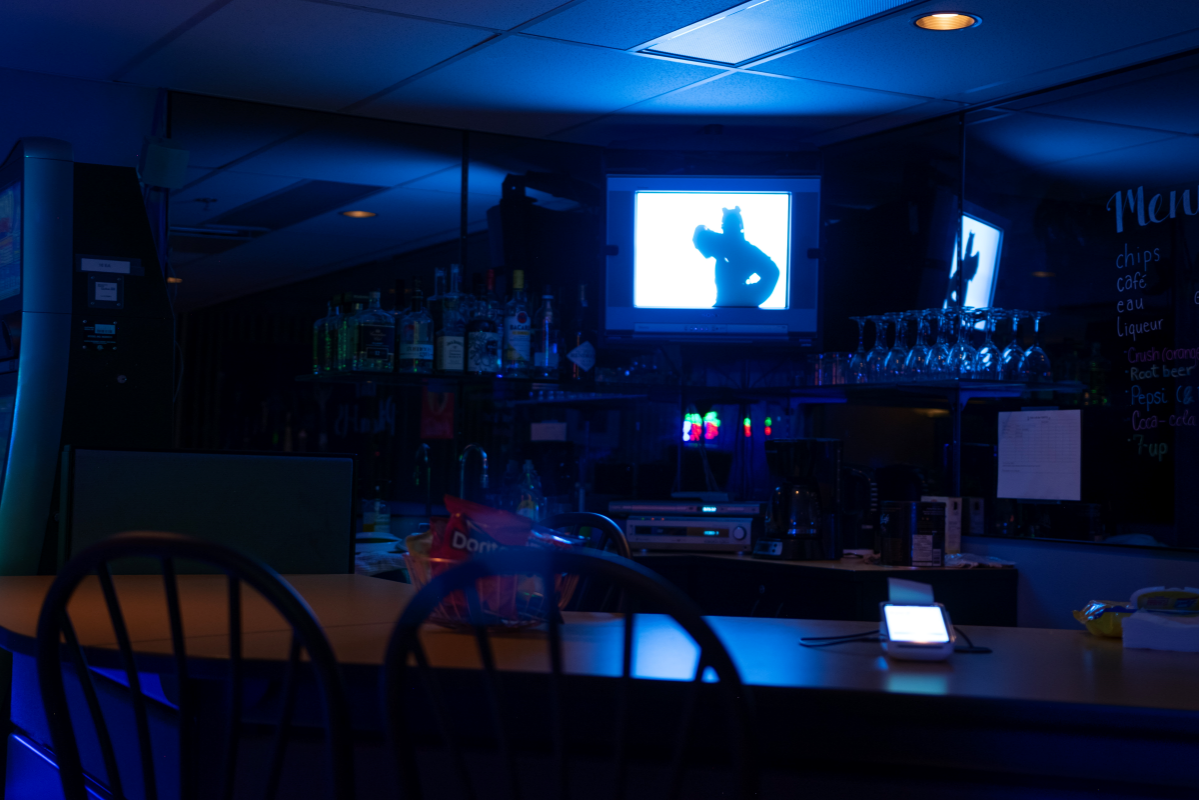
Bar environment—bar paraphernalia and music TV.

Upon entering the bar, the participant is greeted by another member of our research team who plays the role of bartender operating the cash register and ensuring the general safety of the session. The addition of this bartender is done to improve the general realism of the gambling setting. The bar replica has 3 EGMs, but only 1 can be played on and used to show the prevention pop-up messages. The other 2 EGMs are turned on for ambiance purposes, but their money collectors are disabled. Limiting play to only a single EGM eases the recording of gambling behaviors and proper showing of the prevention messages. The participant is told that the 2 unusable EGMs are awaiting servicing due to a malfunction with their money collector.

The participants are free to do as they please (eg, play EGMs, buy chips and nonalcoholic beverages, relax in the bar, etc). They are allowed to leave the bar if they need to go to the bathroom or smoke. They are accompanied by the bartender if they elect to do so. The reference time point of the experiment (ie, T+00:00:00) starts when the participant inserts money for the first time in the EGM. Note that breaks in play, no matter the motive, do not stop the experiment timer regarding the duration limit.

**Figure 6.**
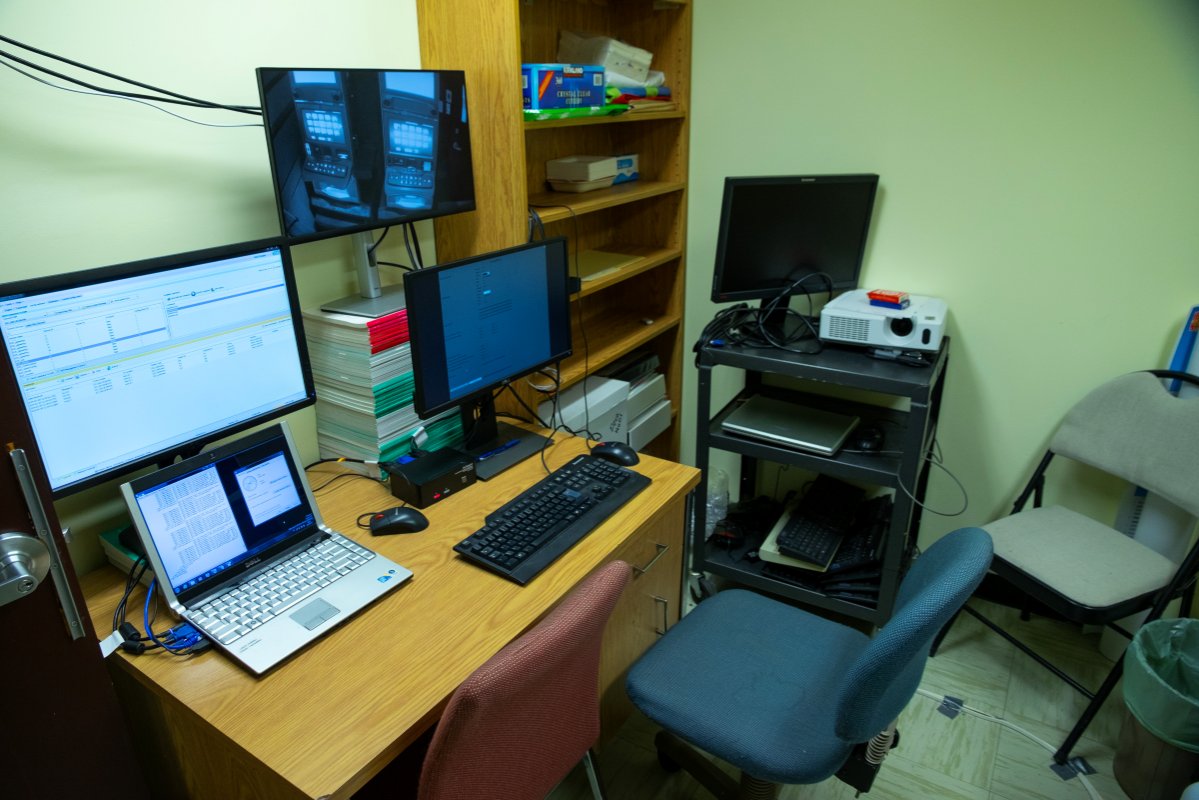
Observation post.

While the participant is in the bar, a second research assistant observes from an adjacent room and is responsible for recording all gambling behaviors using a computer connected to the EGM and operating the pop-up messages presentation schedule (see Figure 6). A live-feed spy camera disguised as a smoke detector is used to observe gambling-related behaviors that cannot be recorded by the EGM’s computer (eg, taking a break).

The session ends when one of the two following conditions is met: (1) the participant expresses, without ambiguity, that they wish to end the experiment, and (2) T+02:00:00 is reached. The procedure may slightly vary depending on how the gambling session ends. Under normal circumstances, the participants end their session by themselves, before reaching the T+02:00:00 time limit. In this scenario, the procedure continues normally, as described in the postexperimentation phase section. Alternative scenarios are:

- Alternative scenario 1: The participant reaches the T+02:00:00 time limit and continues to play. The member of the research team, playing the role of bartender, interrupts the participants and suggests taking a pause to answer some questions. If the participant agrees, the procedure continues normally, as described in the postexperimentation phase section. Unbeknownst to them, they will not be allowed to resume gambling after said questions.
- Alternative scenario 2: Same as “Alternative scenario 1,” but the participant refuses and insists on continuing to play, and debriefing is done on the spot to end the play session. All questionnaires presented in the postexperimentation phase section are done, but contrary to the normal scenario, evaluation of participants’ perception of the realism of the bar replica and of the gambling session is done after debriefing.
- Alternative scenario 3: The participants try to hastily quit the laboratory before we get a chance to debrief them (eg, because they are angry about losing a lot of money and do not wish to participate any longer). In this case, debriefing is done early to prevent them from quitting without receiving all the information to make an informed decision about their participation. All questionnaires presented in the postexperimentation phase section are done, but contrary to the normal scenario, evaluation of participants’ perception of the realism of the bar replica and of the gambling session is done after debriefing.

#### Active Control Group: Regular Gambling Session

This condition is designed to closely replicate a typical gambling session in a natural setting.

##### Electronic Gambling Machines (EGMs)

Gambling is done on fully functional and unaltered EGMs, model IGT GL20. This is the model currently in use in Quebec, Canada, the location where this study is conducted. The wins and losses sequence is fully randomized and not determined beforehand. The payout rate is the same as what is prescribed by law (92%). These machines come equipped with basic responsible gambling features, the 2 main ones being a mandatory time limit reminder and a submenu containing responsible gambling information.

##### Mandatory Time Limit Reminder

**Figure 7.**
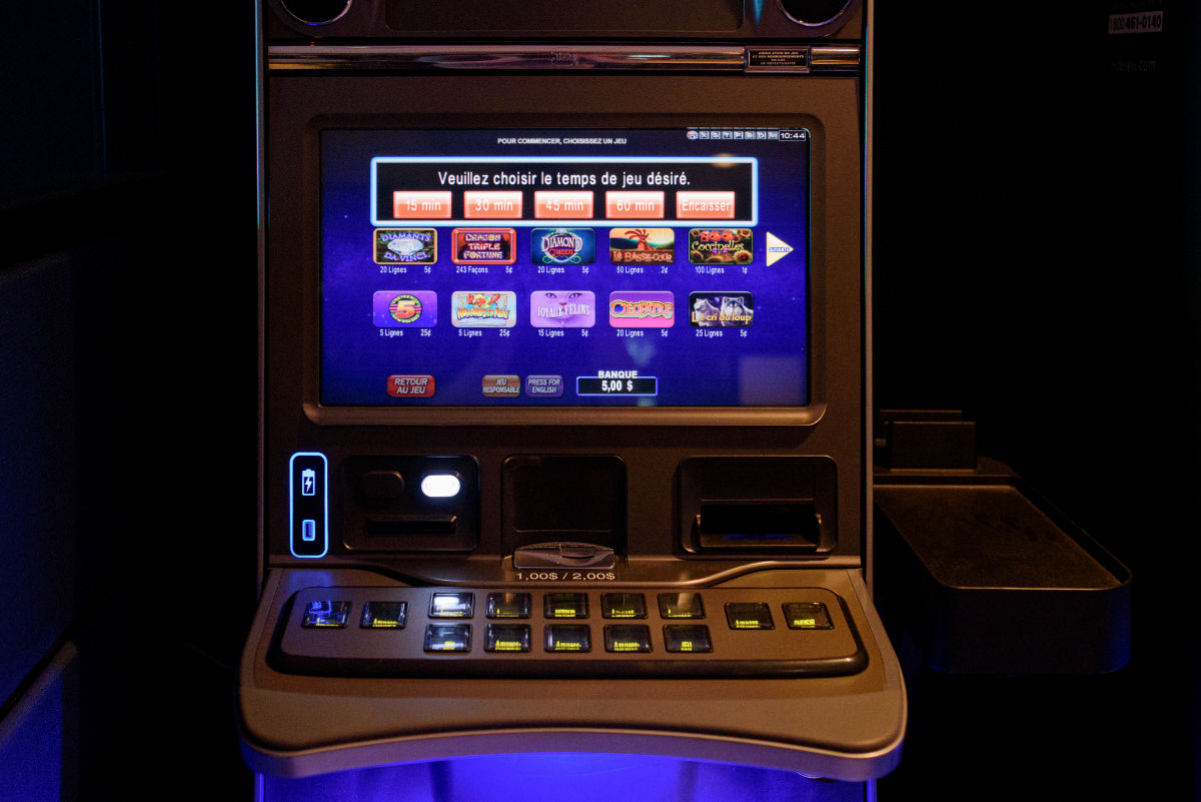
Mandatory time limit reminder.

The mandatory time limit reminder is activated either when (1) the previous time limit is up or (2) the player bank meter reaches CA $0. When the previous time limit is up, a pop-up takes up the upper third of the play screen and requires the player to set a new time limit using one of 5 choice buttons (first four buttons: 15, 30, 45, or 60 minutes; fifth button: cash out, which prints a receipt and ends the gambling session; see Figure 7). As the EGM cannot track who is playing, there are no limits on consecutive gambling sessions. When the player’s bank meter reaches CA $0, the pop-up described above appears as soon as new money is inserted in the EGM, no matter if the previous time limit is up or not.

##### Responsible Gambling Information

**Figure 8.**
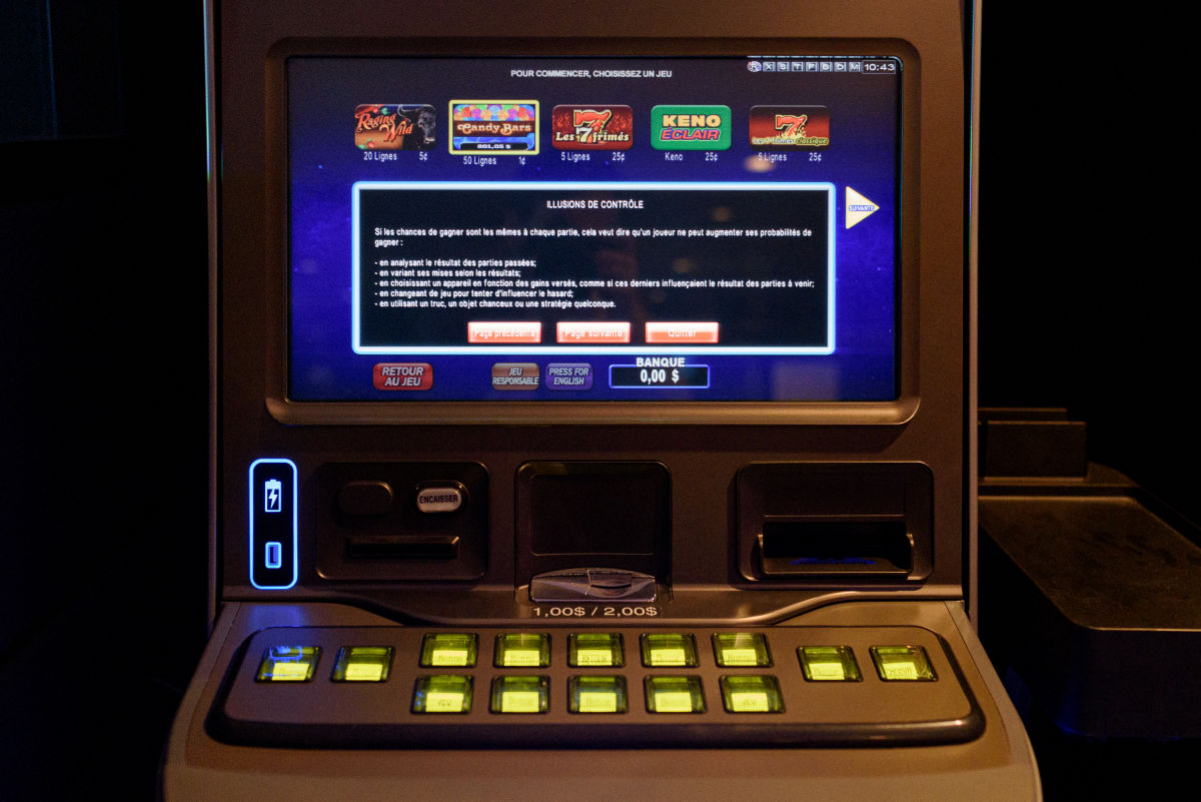
Responsible gambling information.

A submenu accessed through a button on the main touch screen opens a window, which contains basic information on randomness and responsible gambling advice. The information is presented in small white font on a black background (see Figure 8 for an example and Multimedia Appendix 2 for a complete overview).

##### Food Purchases and Money Withdrawals

A payment terminal allows for buying nonalcoholic beverages (soft drinks or coffee) and chips during the experimentation. This is done to enhance the realism of the bar setting. It also allows for real cash withdrawal from a bank account during a gambling session. Quebec’s code of conduct for EGM commercialization prohibits retailers from having an ATM near their EGMs. It also prohibits them from lending money to consumers or withdrawing cash for them to gamble. However, this rule is not always easy to enforce in practice. For this study, participants are allowed to withdraw from their bank account if they ask by themselves. This option is not communicated upfront to them.

### Experimental Group: Regular Gambling Session With Prevention Pop-Up Messages

This condition is the same as the active control group with the addition of prevention pop-up messages delivered during the gambling session on a fixed presentation schedule.

#### Prevention Pop-Up Messages

Messages are shown full screen on the EGM’s play screen on a fixed presentation schedule from the moment the participant inserts money for the first time in the EGM (ie, T+00:00:00). The first message is shown at T+00:10:00. Messages block the play screen for 15 seconds before automatically closing. This presentation frequency is thought to be a good compromise between a very high and potentially annoying frequency (eg, [21,48]) and a very low frequency where only a few participants are exposed (eg, [23]). The first message is presented sooner than the others so that every participant has a decent chance to see at least one. The subsequent presentation schedule is modeled on Schrans et al [50]. While it would have been ideal that messages stay on until participants close them, this was not possible due to hardware limitations on the EGMs’ part. The 15-second presentation duration was chosen based on the mean reading speed of a typical francophone adult [76], so participants have enough time to read the messages.

**Figure 9.**
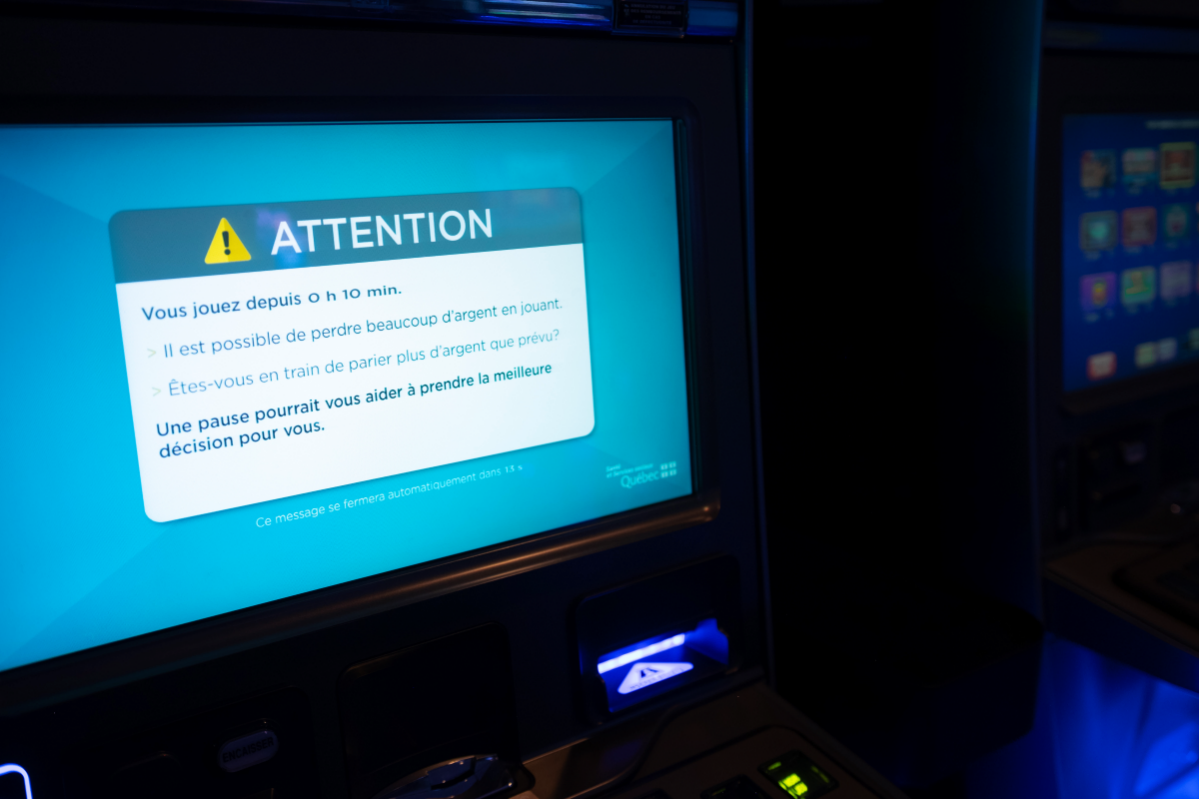
The prevention pop-up message, as displayed on the electronic gambling machine (EGM).

Up to 4 different messages are presented during the gambling session. Order is determined by sortition without replacement. Variations were introduced to counter the possible habituation effect [19,64]. All messages follow the same template (see Textbox 2 and Figure 9 for an example and see Multimedia Appendix 3 for every message used in the study).

Textbox 2. **Prevention pop-up messages template.**

- The message is written in black characters on a white box to maximize text-background contrast.
- The message box is placed on a blue background, which matches the EGMs’ color palette. This is done so that messages appear native to the EGMs, not something introduced for a study. The background pattern behind the message box directs the eye toward the message. The signal word “Attention!” (ie, the French translation for “Caution!”) is used conjointly with a warning pictogram (yellow triangle containing an exclamation mark). The signal word is written in capital letters.
- The bulk of the messages comprised 4 concise statements:

-  The first statement is the time spent gambling since the beginning of the session. This portion is a factual statement to ground the participant in the present.
-  The second statement is a warning that gambling could lead to serious monetary loss. This is a realistic risk associated with gambling. We choose to warn about monetary harms because they are relevant to every gambler (anyone can experience betting too much money, and this can have consequences even after a single session).
-  The third statement is a self-appraisal question about current gambling behavior. This is the only part of the message that changes across versions. There are 4 different variations on this question. Self-appraisal questions drive participants to develop self-determined motivation and goals regarding their gambling behavior.
-  The fourth statement is a simple piece of advice that a break could help choose what is best for oneself. This advice aims to foster participants’ self-efficacy sentiment, enabling them to act in a way to protect themselves [77,78].

- Just below the message is a countdown indicating the time left before the message closes. This is to reassure participants that the message is not a bug of the EGM and that the only thing required of them is to wait and pay attention.
- The province’s Health Department logo is shown in the lower right corner as a more neutral and expert endorsement of the messages. From a general standpoint, the more a source is considered expert and trustworthy, the more a message is susceptible to having an effect [79]. In gambling, Munoz et al [80] found that the participants trusted a source that gave more attention to prevention messages from a perceived independent source (governmental organization) than from a source perceived as having a conflict of interest (org. funded by the local gambling operator). Furthermore, Lemarié and Chebat [81] reported that antigambling ads led to more gambling when coming from a gambling operator.

.

Note that due to hardware limitations on the EGMs’ part, the prevention messages presented only alter what is shown on the play screen (they fill the screen completely, hiding the game), but cannot pause the game per se. The EGMs’ buttons, sound, and music are still operational while the message is shown.

### Postexperimentation Phase

The participant is brought in a separate office after the end of the gambling session for a series of questionnaires (outcomes listed in “Secondary Outcome” and “Other Outcomes” sections) which are, in order:

- Evaluation of participants’ perception of the realism of the bar replica and of the gambling session.
- Evaluation of participants’ ability to recall prevention pop-up messages. This includes multiple tasks of free recall (eg, describing the pop-up messages from memory) and cued recall (eg, selecting from a list the pop-up messages that were seen during the gambling session).
- Evaluation of participants’ cognitive and emotional response to prevention pop-up messages.
- Evaluation of protocol credibility (ie, if the participant really believed they were gambling their own money and were really risking their own money).

These questionnaires are comprised of Likert scales, multiple-choice questions, and open-ended questions. Contrary to more standard qualitative designs, open-ended question responses are not recorded in an audio format, nor are they transcribed verbatim [82]. Instead, the interviewer takes notes, which the participant can see on a computer screen. Participants are then asked to confirm that the interviewer’s notes accurately reflect their opinion. It has been suggested that note-taking is superior to audio recording for obtaining qualitative data [83].

Debriefing about the true goals of the study and final validation of consent to participate in the study are done between the evaluation of participants’ perception of the realism and the evaluation of participants’ ability to recall prevention pop-up messages.

### Outcome Assessments

Table 3 shows the primary outcome measures.

**Table 3.** Primary outcome measures.

Outcome category	Outcome number	Outcome	Description	Measurement time points
Gambling behavior: Money				
	1	Money betted (physical)	Cumulative amount of money physically inserted in the EGM^a^ during the gambling session.	Assessed at the following time points from start of gambling session in the laboratory: [0] T+00:00:00, [1] T+00:10:00, [2] T+00:40:00, [3] T+01:10:00, [4] T+01:40:00, [5] end of gambling session (max T+02:00:00)
	2	Change in money betted (physical)	Change between self-reported measures (past 12 months) and observed data in the laboratory.	Past 12 months; end of the gambling session
Gambling behavior: Time				
	3	Gambling session’s total duration	Time elapsed between the first time the participant puts money in the EGM (T+00:00:00) to the end of the gambling session. Session’s duration is left for the participant to decide. While the participant is told they can play for as long as they like, there is a 2 h limit on the session duration. This limit is hidden from the participant. Session’s duration includes any breaks taken from gambling on the EGM.	Assessed at the end of the gambling session.
	4	Total time effectively spent gambling	Time elapsed between the first time the participant puts money in the EGM (T+00:00:00) to the end of the gambling session, minus breaks taken from gambling on the EGM.	Assessed at the end of the gambling session.
	5	Change in total time effectively spent gambling	Change between self-reported measure (past 12 months) and observed data in the laboratory	Past 12 months; end of the gambling session.
Gambling behavior: Breaks				
	6	Number of breaks taken	Number of breaks taken from gambling on the EGM during the gambling session.	Assessed at the end of the gambling session.
	7	Mean breaks’ duration	Average duration of breaks taken from gambling on the EGM over the gambling session’s total duration.	Assessed at the end of the gambling session.
	8	Total breaks duration	Sum of breaks’ duration taken from gambling on the EGM over the gambling session’s total duration.	Assessed at the end of the gambling session.
	9	Mean time elapsed between breaks	Average time separating any two breaks taken from gambling on the EGM over the gambling session’s total duration.	Assessed at the end of the gambling session.
Gambling behavior: Risk taking				
	10	Number of bets placed	Number of bets placed on the EGM during the gambling session.	Assessed at the following time points from start of the gambling session in the laboratory: [0] T+00:00:00, [1] T+00:10:00, [2] T+00:40:00, [3] T+01:10:00, [4] T+01:40:00, and [5] at the end of the gambling session (max T+02:00:00).
	11	Gambling speed	Number of bets placed on the EGM during the gambling session over a given amount of time (ie, bets/min).	Assessed at the following time points from start of the gambling session in the laboratory: [0] T+00:00:00, [1] T+00:10:00, [2] T+00:40:00, [3] T+01:10:00, [4] T+01:40:00, and [5] at the end of the gambling session (max T+02:00:00).
	12	Gambling intensity (physical)	Money physically inserted in the EGM during the gambling session over a given amount of time (ie, $/min).	Assessed at the following time points from the start of the gambling session in the laboratory: [0] T+00:00:00, [1] T+00:10:00, [2] T+00:40:00, [3] T+01:10:00, [4] T+01:40:00, and [5] end of the gambling session (max T+02:00:00).
	13	Gambling intensity (all)	Money betted on the EGM during the gambling session over a given amount of time (ie, $/min). This is money used to “buy rounds on the EGM,” whether it's money physically inserted in the EGM or money won while gambling and betted again.	Assessed at the following time points from the start of the gambling session in the laboratory: [0] T+00:00:00, [1] T+00:10:00, [2] T+00:40:00, [3] T+01:10:00, [4] T+01:40:00, [5] end of the gambling session (max T+02:00:00).
Cognitions				
	14	Perceived self-control while gambling on EGMs	Participant’s perceived ability to control their gambling behavior (eg, sticking to their predetermined limits) over the gambling session’s total duration. Evaluated with a 7-point Likert scale (1 = “I never had control” to 7 = “I always had control”).	Assessed at the end of the gambling session.
	15	Change in perceived self-control while gambling on EGMs	Change between self-reported measure (past 12 months) and observed data in the laboratory	Past 12 months; end of the gambling session.
	16	Perceived behavioral effectiveness of prevention pop-up messages	Participant’s perceived effectiveness of prevention pop-up messages on modifying their gambling behavior during the gambling session. Evaluated with two 7-point Likert scales about the perceived effects of the prevention pop-up messages on money betted and time spent during the gambling session (1=“lowered gambling behavior a lot” to 7=“heightened gambling behavior a lot”).	Assessed at the end of the gambling session.
Emotions				
	17	General level of fun while gambling on EGMs	Participant’s general enjoyment of gambling on EGMs over the gambling session’s total duration. Evaluated with a 7-point Likert scale (1=“I hated it” to 7=“I loved it”).	Assessed at the end of the gambling session.
	18	Change in general level of fun while gambling on EGMs	Change between self-reported measures (past 12 months) and observed data in the laboratory.	Past 12 months; end of the gambling session.
	19	Psychological reactance to prevention pop-up messages (between group)	Evaluated based on Dillard and Shen [84] method: (1) Induction check (4 items, 7-point Likert scale, 1=“Strongly disagree, 7=“Strongly agree”); (2) Anger (4 items, 7-point Likert scale, 1=“Strongly disagree, 7=“Strongly agree”); (3) Cognitive response (count of negative thoughts in relation to the pop-up messages); and (4) Attitude (7 word pairs, 7-point semantic differential scale).	Assessed at the end of the gambling session.

a EGM: electronic gambling machine.

#### Secondary Outcome

Table 4 shows the secondary outcome measures.

**Table 4.** Secondary outcome measures.

Outcome category	Outcome number	Outcome	Description	Measurement time points
Feasibility: Recruitment potential
	20	Volunteers	Number of people that answered the recruitment ad and wanted to either participate or get more information on the study.	N/A^a^
	21	Recruited	Number of people that qualified and agreed to participate in the study.	N/A
	22	Rejected	Number of people that volunteered to participate but did not qualify according to the inclusion and exclusion criteria.	N/A
	23	Refusal	Number of people that qualified for participation in the study but refused to do so after hearing the details of it.	N/A
	24	Refusal reasons	N/A	N/A
	25	Attrition	Number of people that qualified and agreed to participate in the study but desisted during their participation.	N/A
	26	Attrition vs previous study	Conversion from recruitment list from a previous study	N/A
Feasibility: Perceived realism
	27	Gambling location type most resembling the session in the laboratory	Among a predetermined list of gambling location type (eg, bar, restaurant, casino, etc), the participant chose which of the gambling sessions in the laboratory resembled the most.	Assessed at the end of the gambling session
	28	Session in the laboratory versus gambling on EGMs in a bar/restaurant	Level of resemblance between a gambling session on EGMs^b^ in a bar or restaurant and the gambling session in the laboratory. Evaluated with a 7-point Likert scale (1=“Almost 100% different” to 7=“Almost 100% the same”).	Assessed at the end of the gambling session
	29	Similarities between sessions in the laboratory versus gambling on EGMs in a bar/restaurant	Things that were the same or very alike when comparing a gambling session on EGMs in a bar or restaurant and the gambling session in the laboratory. Participant lists all the resemblances that come to their mind.	Assessed at the end of the gambling session
	30	Differences between session in the laboratory versus gambling on EGMs in a bar/restaurant	Things that were not the same or not very alike when comparing a gambling session on EGMs in a bar or restaurant and the gambling session in the laboratory. Participant lists all the differences that come to their mind.	Assessed at the end of the gambling session
	31	What could be done to heighten the realism of the session in the laboratory	Things or aspects of the study that could be changed in order for the gambling session in the laboratory to be more like a real gambling session on EGMs in a bar or restaurant. The participant lists all that come to their mind.	Assessed at the end of the gambling session
	32	Effect of using one's own money on realism	Effect of gambling one’s own money in this study on the level of realism. Evaluated with a 7-point Likert scale (1=“It lessened the realism a lot” to 7="It heightened the realism a lot").	Assessed at the end of the gambling session
	33	Effect of using one's own money on realism (open-ended)	Effect of gambling one’s own money in this study on the level of realism. Participants freely explain their perception of the said effect.	Assessed at the end of the gambling session
	34	Perceived correspondence between habitual gambling behavior and gambling behavior during the session in the laboratory	Participant’s perception of how much their gambling behavior during their gambling session in the laboratory was representative (ie, how much it was the same) of their gambling behavior on EGMs in the past 12 months. Evaluated with a 7-point Likert scale (1=“Almost 100% different” to 7=“Almost 100% the same”).	Assessed at the end of the gambling session
	35	Perceived correspondence between habitual gambling behavior and gambling behavior during the session in the laboratory (open-ended)	Participants’ perception of how much their gambling behavior during their gambling session in the laboratory was representative (ie, how much it was the same) of their gambling behavior on EGMs in the past 12 months. Participants freely explain their perception.	Assessed at the end of the gambling session
Feasibility: Duplicity check
	36	Protocol credibility	Participant’s level of certitude when they were betting their own money and that they could lose money for real during the study. Evaluated with a 7-point Likert scale (1=“Almost 100% certain they were going to get their money back at the end of the study” to 7=“Almost 100% certain winnings and losses were real”).	Assessed at the end of the gambling session
	37	Protocol credibility (open-ended)	Participants comment freely on their level of certitude that they were betting their own money and that they could lose money for real during the study.	Assessed at the end of the gambling session

a N/A: not available.

b EGM: electronic gambling machine.

#### Other Outcomes

Table 5 shows other outcome measures.

**Table 5.** Other outcome measures.

Outcome category	Outcome number	Outcome	Description	Measurement time points
Messages recall
	38	Free recall (yes or no)	Participant is asked if they saw any prevention pop-up messages during their gambling session in the laboratory. This is a yes or no question.	Assessed at the end of the gambling session
	39	Free recall (number)	If the participant declared that they saw prevention pop-up messages during their gambling session in the laboratory, they are asked how many there were. The same message presented twice counts as “2”.	Assessed at the end of the gambling session
	40	Free recall (content)	If the participant declared they saw prevention pop-up messages during their gambling session in the laboratory, they are asked to freely describe them (ie, content, graphical appearance, etc).	Assessed at the end of the gambling session
	41	Cued recall (yes or no)	The participant is presented with a list of prevention pop-up messages that could have been shown during the study. For each of them, they are asked to identify if they saw it or not.	Assessed at the end of the gambling session
	42	Cued recall (number)	If the participant declared that they saw prevention pop-up messages during their gambling session in the laboratory, they are asked how many there were. The same message presented twice counts as “2”. This is different from outcome 39 because the participant might recognize a message they had forgotten about or a message they saw but did not consider it a “prevention message.”	Assessed at the end of the gambling session

### Statistical Methods

Sociodemographic variables, past 12 months of gambling-related variables, and PGSI category will be presented in a table with appropriate central tendency estimates. This table will be divided by assignation group.

A total of 3 statistical models will be used to analyze the outcomes presented in Tables3  4. All these models will control for the time-varying covariate “gains-losses differential” (ie, remaining balance on the EGM at measured time point minus money inserted in the EGM from the start of the study). All models will also test the moderation effect of the following variables: gender (men or women), age (continuous), education (5 categories), and PGSI category (nonproblem, low risk, and moderate risk). Holm-Bonferroni correction [85] will be used to counteract family-wise error rate due to multiple comparisons in each model for each statistically significant categorical variable (ie, time for repeated measures ANCOVAs; moderators with ≥3 categories).

The first model uses ANCOVAs to examine the differences between the two groups at the end of the gambling session on outcome variables 1, 3, 4, 6‐9, 14, 16, 17, and 19, separately.

The second model uses repeated measures ANCOVAs to examine the differences between the two groups at specific time points during the experiment corresponding to the presentation of the prevention pop-up messages in the experimental group. This model is computed on outcome variables 1 and 10‐13, separately.

The third model uses repeated measures ANCOVAs to examine the differences between self-reported measures (past 12 months) and the observed behaviors during the experiment. This model is computed on outcome variables 2, 5, 15, and 18, separately.

Secondary and other outcomes will be documented but will not be statistically compared. Descriptive estimates will be provided for each quantitative variable. Open-ended questions will be analyzed in a manner inspired by the thematic analysis method (see Textbox 3) [86].

Textbox 3. **The 5 steps in analysis of open-ended questions.**

1. Interviewer’s notes are regrouped in a Microsoft Word document.
2. Raw data is carefully read, both by main author and another member of the research team, to extract categories of similar content.
3. Categories are refined by both coders.
4. Raw data is independently categorized in appropriate categories by both coders.
5. Intercoder agreement is calculated on all categorizations to ensure reliability.

## Results

Recruitment began in February 2024 and concluded in December 2024. Results are expected to be published in 2026. No results are currently available.

## Discussion

### Principal Findings

The main objective of this study is to test the potential of pop-up messages as a prevention measure in a gambling setting. Effects of messages are measured on behaviors, cognitions, and emotions. The optimal benefit from the messages would be observing a reduction in money bet during the gambling session. However, other behavioral effects, like reduction in gambling intensity or session lengths, are considered indirectly beneficial because they foster conditions for further preventive actions. For example, all things being equal, a reduction in gambling intensity reduces the amount of money lost per unit of time. This, in turn, gives more time to the person gambling to think about their behavior and potentially stop their session with some money left. The improved realism of our research design heightens the confidence that the effects observed in our study can successfully be transposed to a real gambling setting.

Similarly, cognitive and emotional responses to the pop-up messages are of interest for the ease of adoption and indirect protection. Modifying cognitions is among the first steps in initiating behavioral change. Even if exposure to pop-up messages does not cause immediate reduction in gambling behavior, it is possible that they initiate a reflection about one’s gambling habits. This may lead to behavioral change at some point in the future or make the person more amenable to other gambling prevention strategies. Another aspect to consider regarding cognitive and emotional response to pop-up messages is psychological reactance, which is “the motivational state that is hypothesized to occur when a freedom is eliminated or threatened with elimination” [87]. In essence, the theory of reactance posits that, when a person’s freedom is threatened or removed (eg, being exposed to an RG pop-up message they feel is telling them how to feel or to act), they are motivated to restore it, whether directly by rebelling against the instructions (eg, gambling more) or indirectly, where the rebellion is done cognitively (eg, derogating the source of the message, denying there is even a threat, etc) [84,87-89]. Measuring how people cognitively and emotionally react to the pop-up messages is useful for understanding their effect. Hypothetical negative results could be due to psychological reactance. Data collected in this study, namely open-ended questions, could help understand how to better design pop-up messages to reduce psychological reactance. Furthermore, observing that pop-up messages do not negatively affect the participants’ level of pleasure could be a good argument to reduce potential reticence of the industry to broaden their usage [90].

Finally, this study will generate valuable data regarding the feasibility of studies conducted in a laboratory setting with the use of real money. Results will help determine if concerns are founded regarding participants’ potential uneasiness with gambling their own money in the laboratory and choosing not to participate in the study. Furthermore, results will show if the use of duplicity was successful in making participants believe they were really at risk of losing money. Feedback from participants could help improve this aspect if necessary.

### Comparison With Previous Work

While this study diverges from Auer and Griffiths [28] on certain theoretical underpinnings of the pop-up messages, their conceptualizations are mostly similar. This study has the advantages of measuring more thoroughly the effect of pop-up messages. This is reflected in the fact that this study records individual performance instead of a collection of gambling sessions (with the possibility that some of them have been done by the same individual). Furthermore, this study incorporates a variety of indicators, behavioral and psychological, whereas Auer and Griffiths [28] only evaluate the effect on ending of the gambling session immediately after the apparition of the pop-up messages.

This study also improves on other studies conducted in a real gambling setting using objective observed behavioral data instead of self-reported ones [29,34,49,50]. This limits the potential imprecision associated with the recollection of past events.

Finally, this study innovates by the level of ecological realism of the main task. To our knowledge, no other study conducted in the laboratory has so closely replicated the conditions of a real gambling setting.

### Strengths and Limitations

This study’s main strengths are the use of a highly realistic setting for the gambling session and the use of duplicity to make participants believe that they are risking their own money. Furthermore, the maximal possible gain (CA $500) is sufficiently high to elicit an incentive to gamble seriously even though the (perceived) level of risk. This level of realism has seldom been achieved in a laboratory setting. Another strength is the diversity of outcomes observed. More specifically, the use of open-ended questions on multiple aspects heightens the chance to be able to interpret unexpected results.

On the other hand, limitations include potential difficulties in recruiting enough participants. First, regular EGM gamblers do not constitute a relatively small population in which this study is conducted. Furthermore, being a regular EGM player significantly overlaps with probable pathological gambling [91,92], which the ethics committee prohibits us from recruiting. The exclusion of probable pathological gamblers could affect the generalizability of the results. While pathological gamblers would be better served by more intensive prevention measures (or even therapy), they could still theoretically benefit from RG pop-up messages. Therefore, if this study goes well, it would be pertinent to repeat it while including this vulnerable population. In the meantime, it is possible that the pop-up messages observed in this study are higher than if deployed in a real setting due to the exclusion of pathological gamblers.

A second limitation is the impossibility of programming the same sequence of gains and losses for all participants. This is due to hardware limitations on the EGM. While randomness should provide balance between both groups, there is always the possibility that one group is significantly “luckier” than the other, altering how they respond to RG pop-up messages [45,93]. This could limit our ability to observe an effect. The gains-losses differential (see the “Statistical methods” section) is also used as a covariate in the analysis to mitigate this potential source of bias.

Third and finally, it is possible that the protection effect from pop-up messages is best observed over repeated exposure over multiple sessions. Therefore, a single gambling session as used in this study might not be enough to observe the full effect. Nevertheless, short of committing to operate a real gambling venue, there are no real feasible solutions to the “single session” in a laboratory setting with the use of real money. Indeed, duplicity works so long as participants do not know they will be reimbursed for their losses. In a study setting, it is not feasible to keep their money longer than a single session because (1) a participant might not come back for another session and be unreachable, making it impossible for the researchers to give them their money; and (2) even assuming all participants stay engaged in the study to the end, some of them might incur financial hardship that is not compensated by recuperating their money at a later date (eg, a participant might need to pay their rent now, a problem not fixed by getting their money back after months).

### Conclusion

This study will provide new insights on the efficacy of pop-up messages as a prevention measure for gambling as well as the appropriateness of laboratory studies as a substitute to a real-life setting.

## Supplementary material

10.2196/75068 Multimedia Appendix 1 Bar environment.

10.2196/75068 Multimedia Appendix 2 Responsible gambling information.

10.2196/75068 Multimedia Appendix 3 Prevention pop-up messages.

## Abbreviations

EGM: electronic gambling machines
PGSI: Problem Gambling Severity Index
RG: responsible gambling
