# Supplementary figures and images for: Effects of Prevention Messages for Electronic Gambling Machines on Behaviors and Cognitions: Protocol for a Two-Arm Stratified Block: Randomized Controlled Study
Source: JMIR Res Protoc. 2025 Nov 10;14:e75068. doi: 10.2196/75068 (PMC12599998; doi:10.2196/75068)

# EGMs

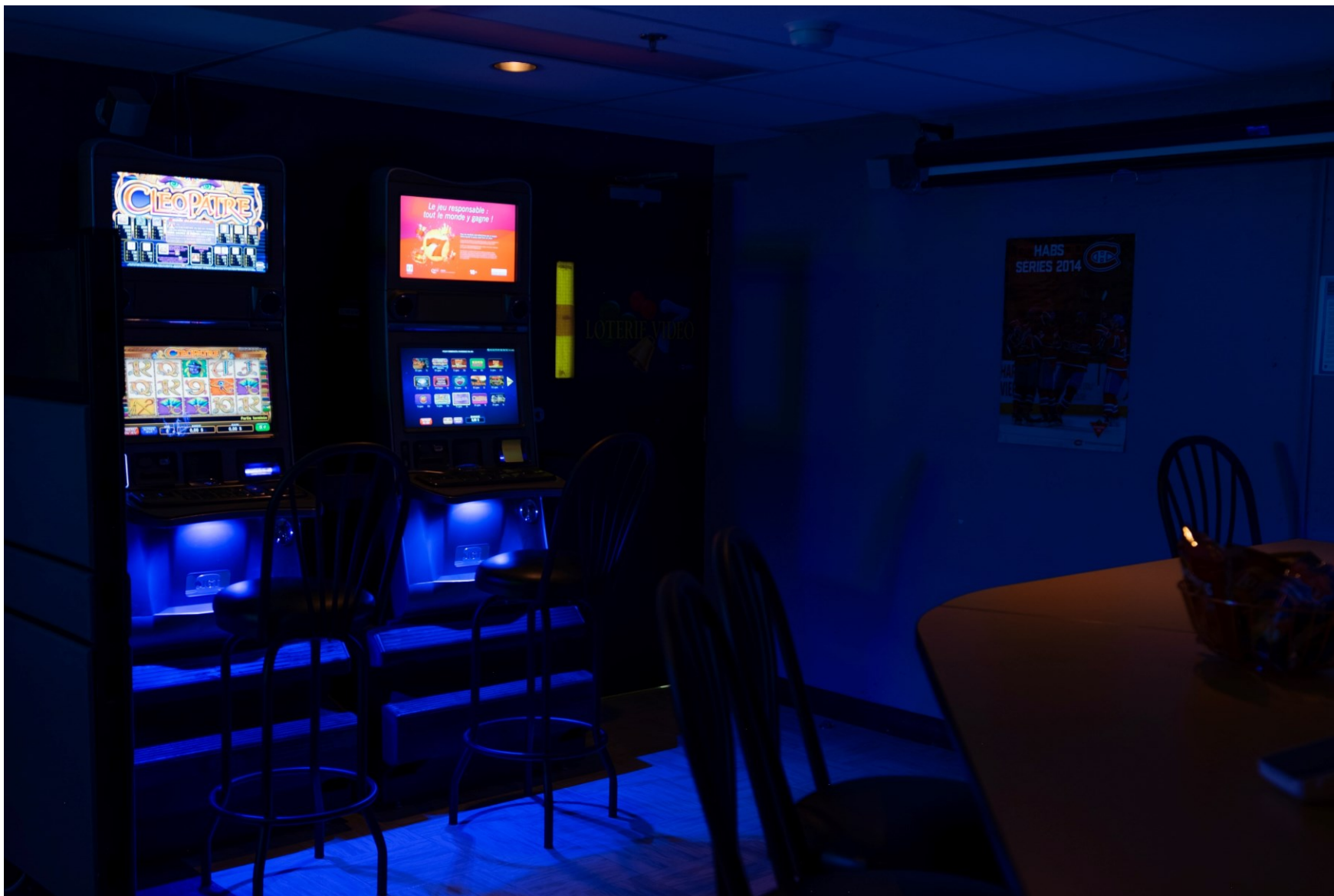

## EGMs – Keypad

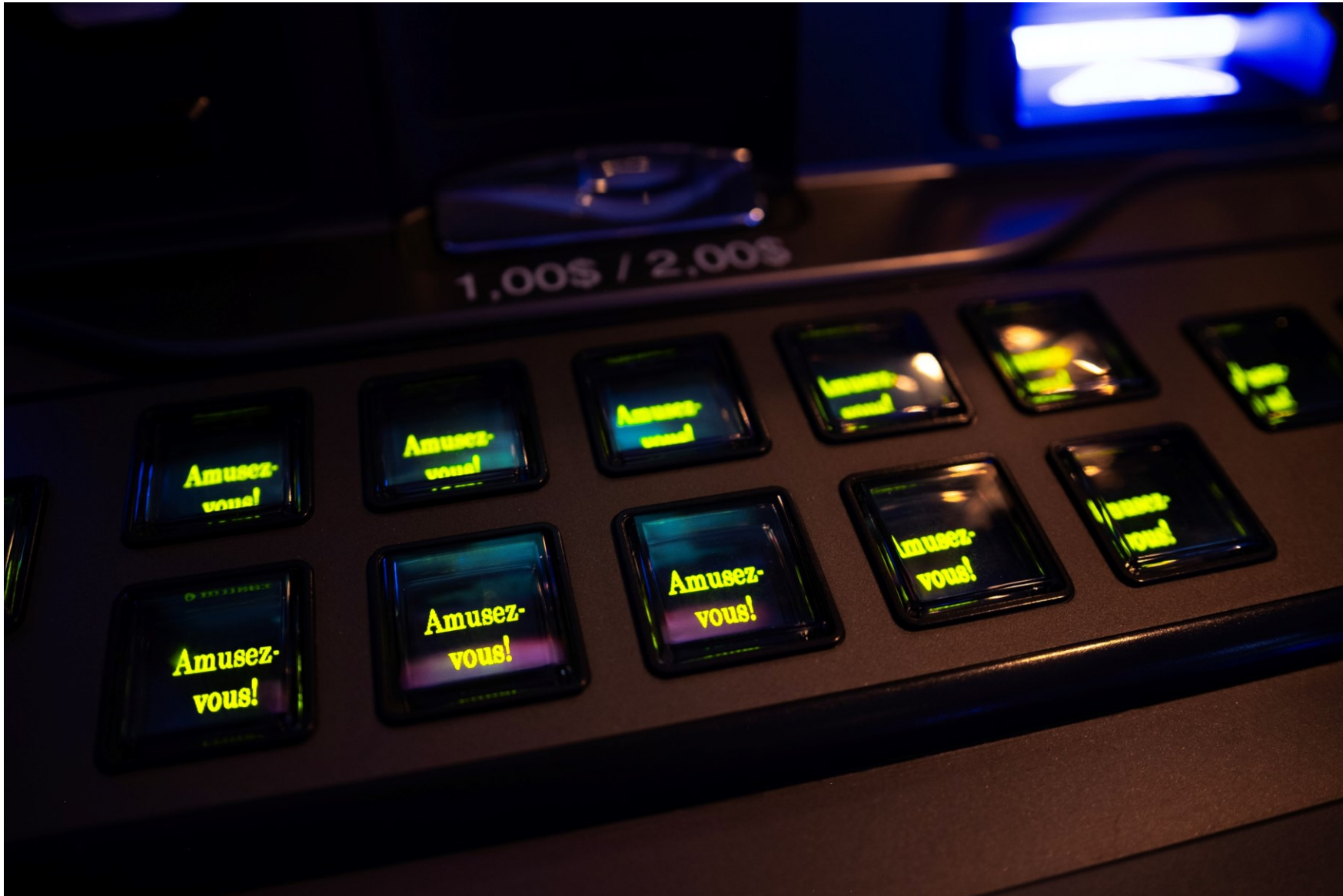

Bar environment – Bar's name on the mirror

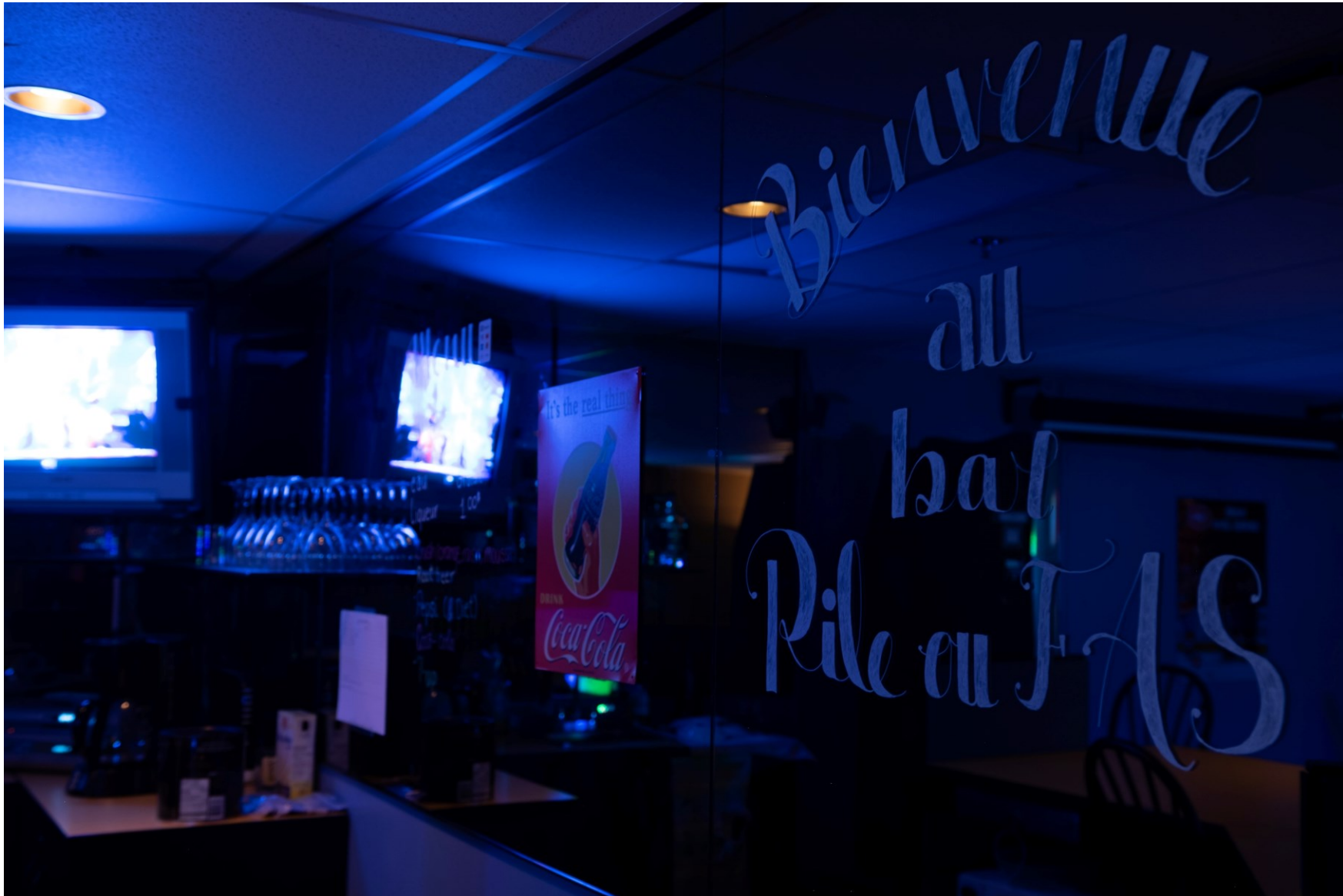

# Square payment terminal

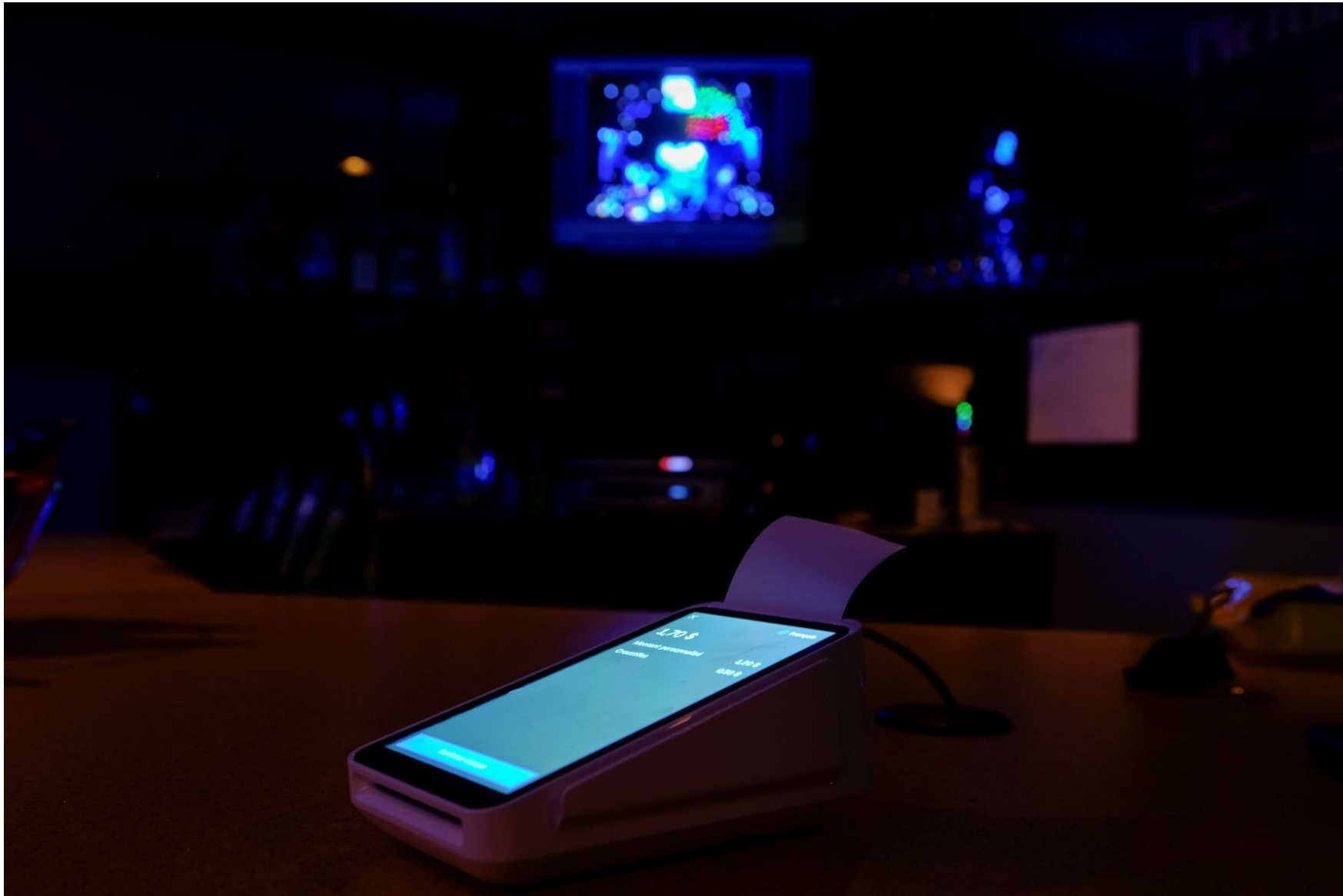

Supplement: Multimedia Appendix 1 [file resprot-v14-e75068-s001.pdf]
