# Supplementary material for: Effects of Prevention Messages for Electronic Gambling Machines on Behaviors and Cognitions: Protocol for a Two-Arm Stratified Block: Randomized Controlled Study
Source: JMIR Res Protoc. 2025 Nov 10;14:e75068. doi: 10.2196/75068 (PMC12599998; doi:10.2196/75068)

# Responsible gambling information – Randomness

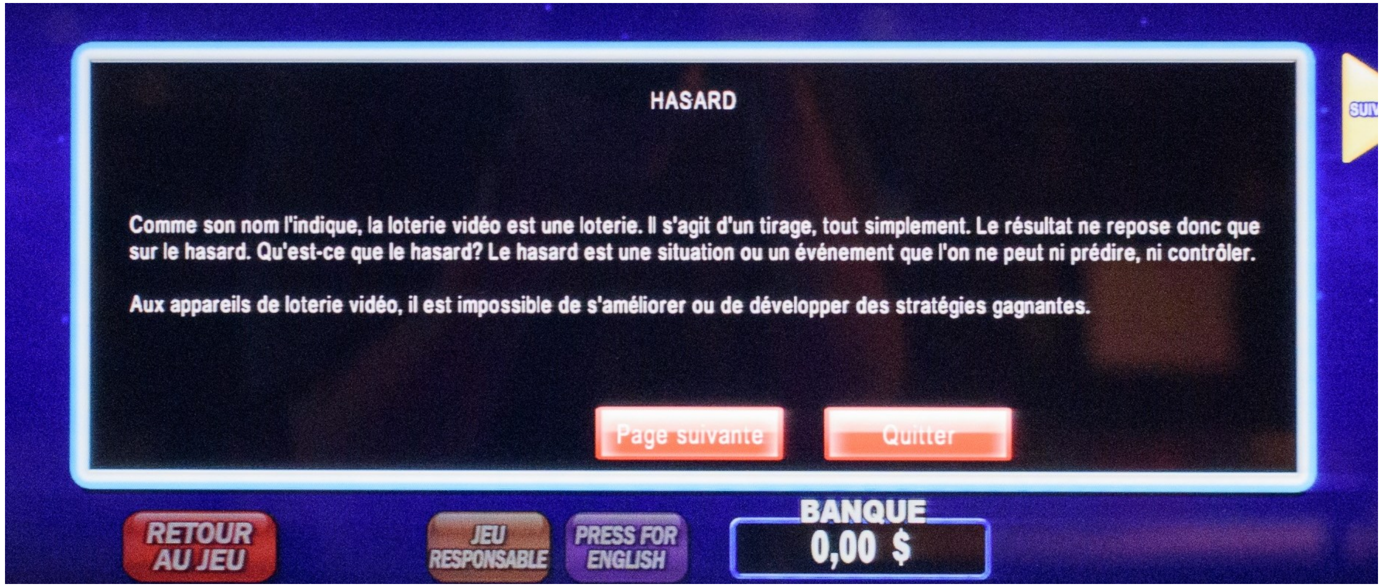


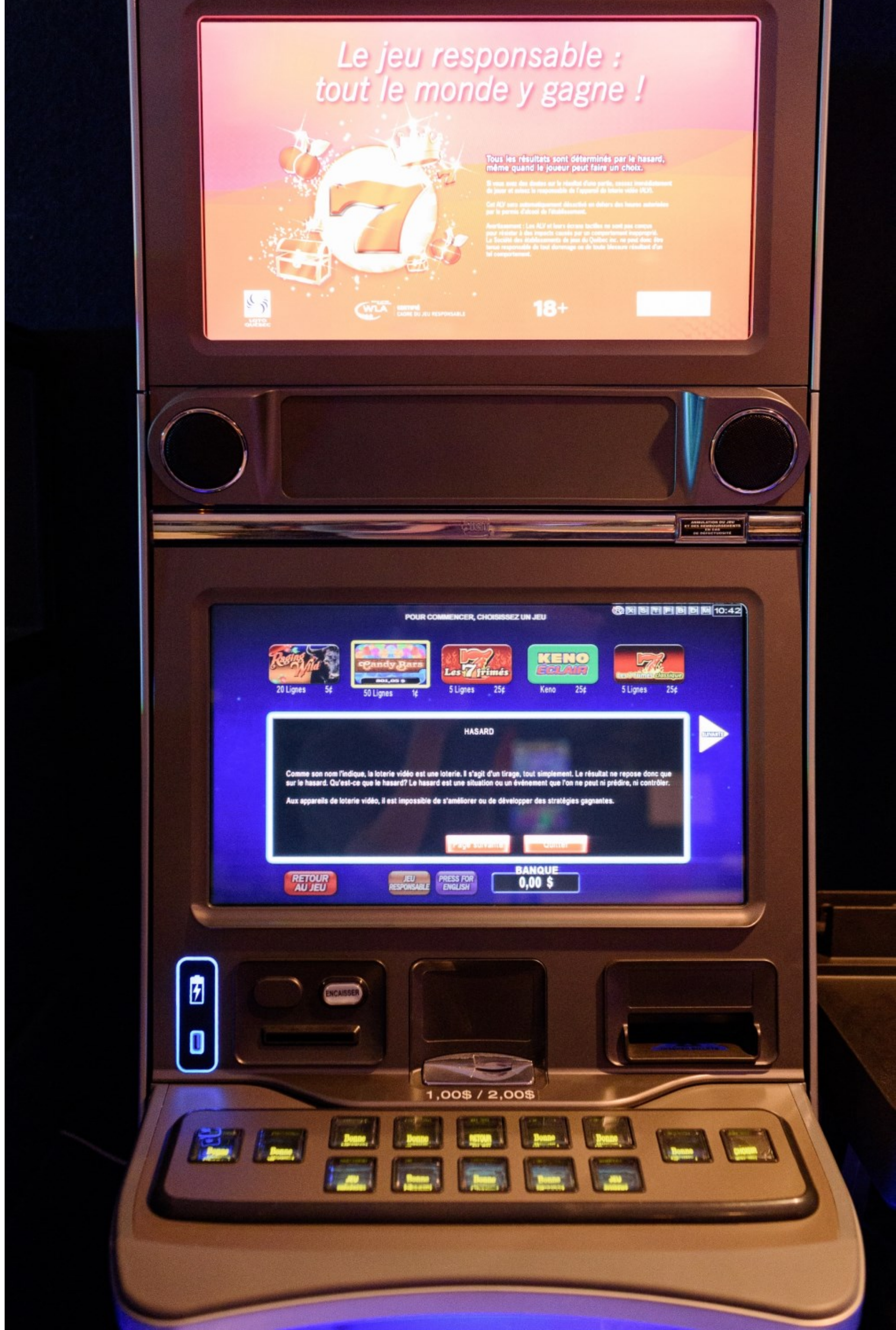

# Responsible gambling information – Illusion of control

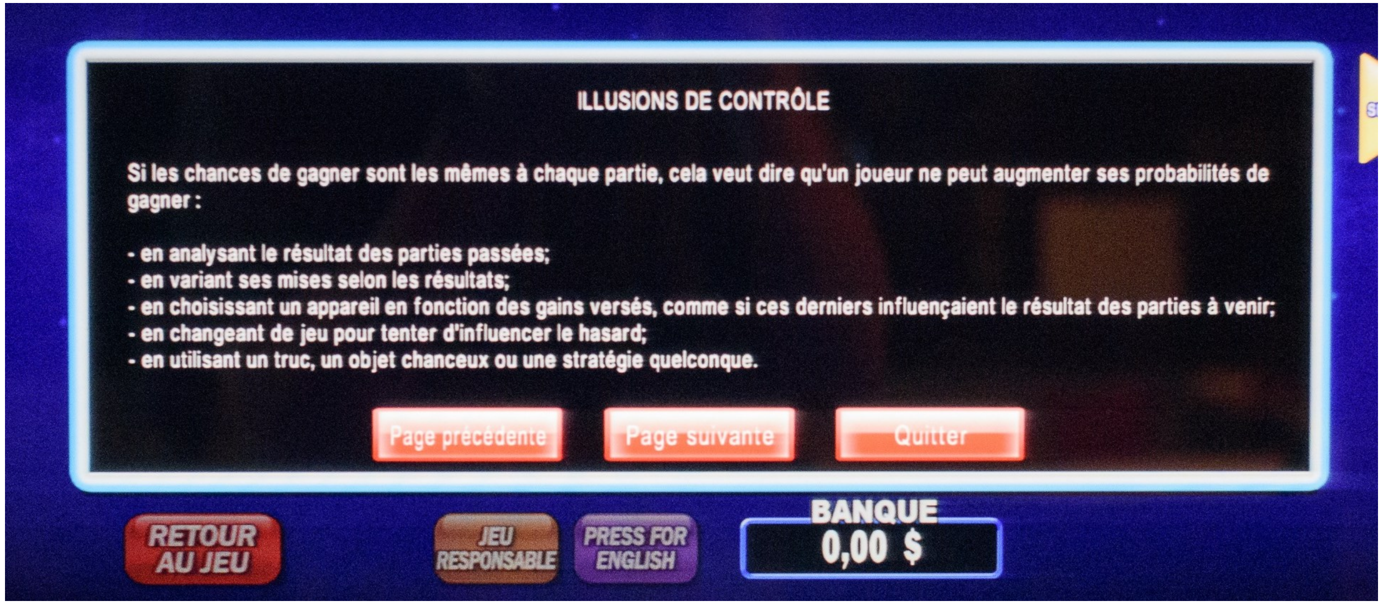


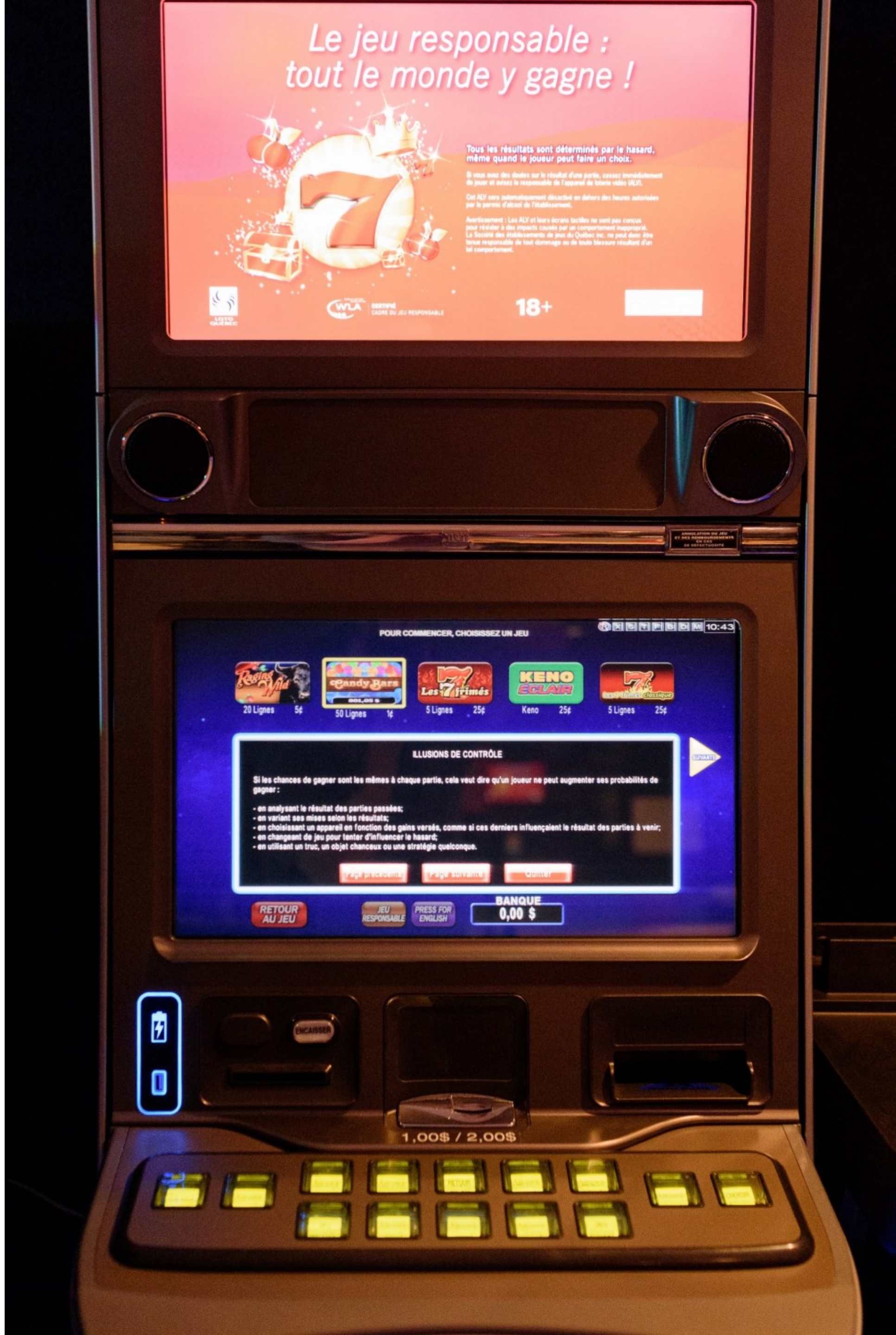

# Responsible gambling information – Independence of events

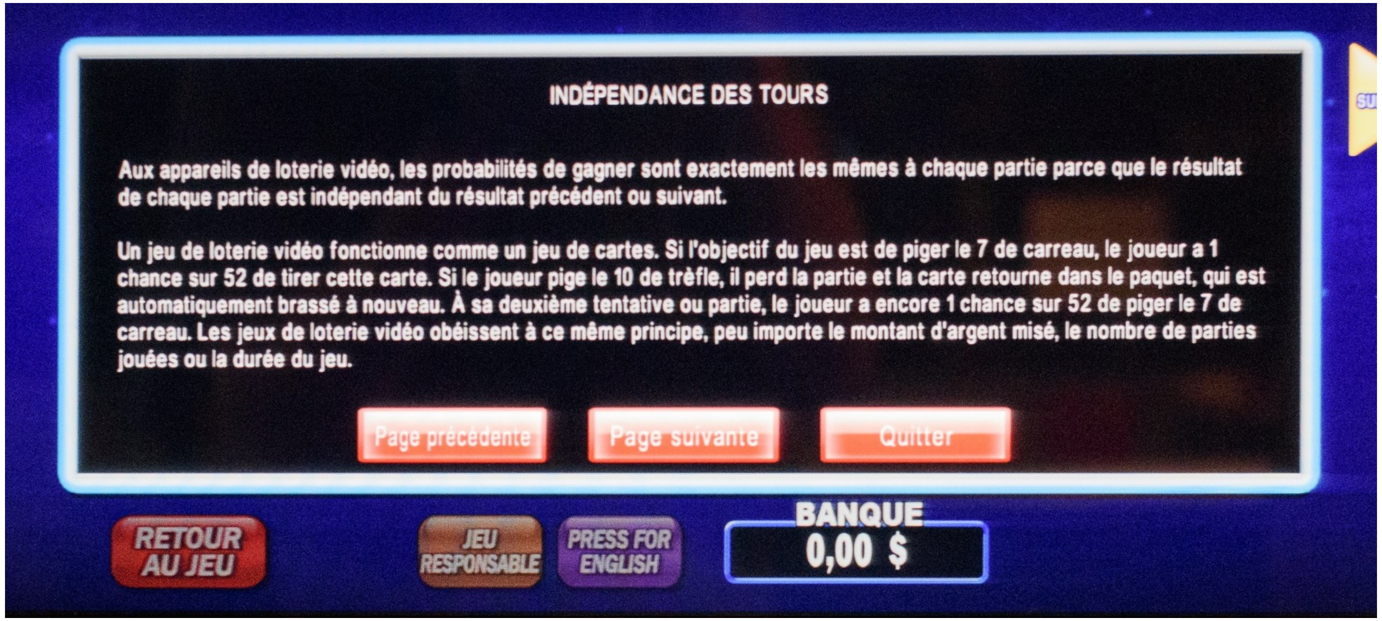


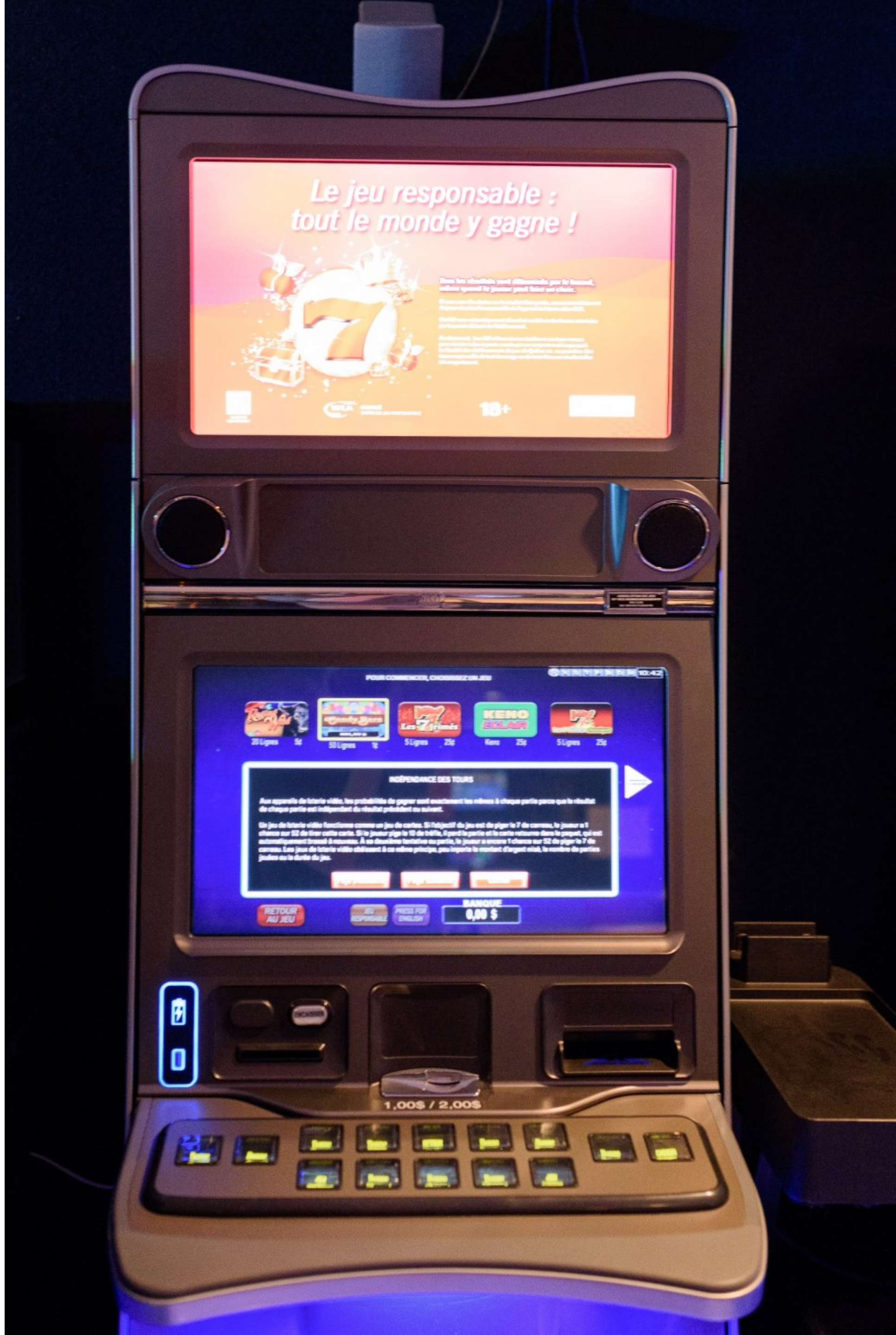

# Responsible gambling information – Knowing when to seek help

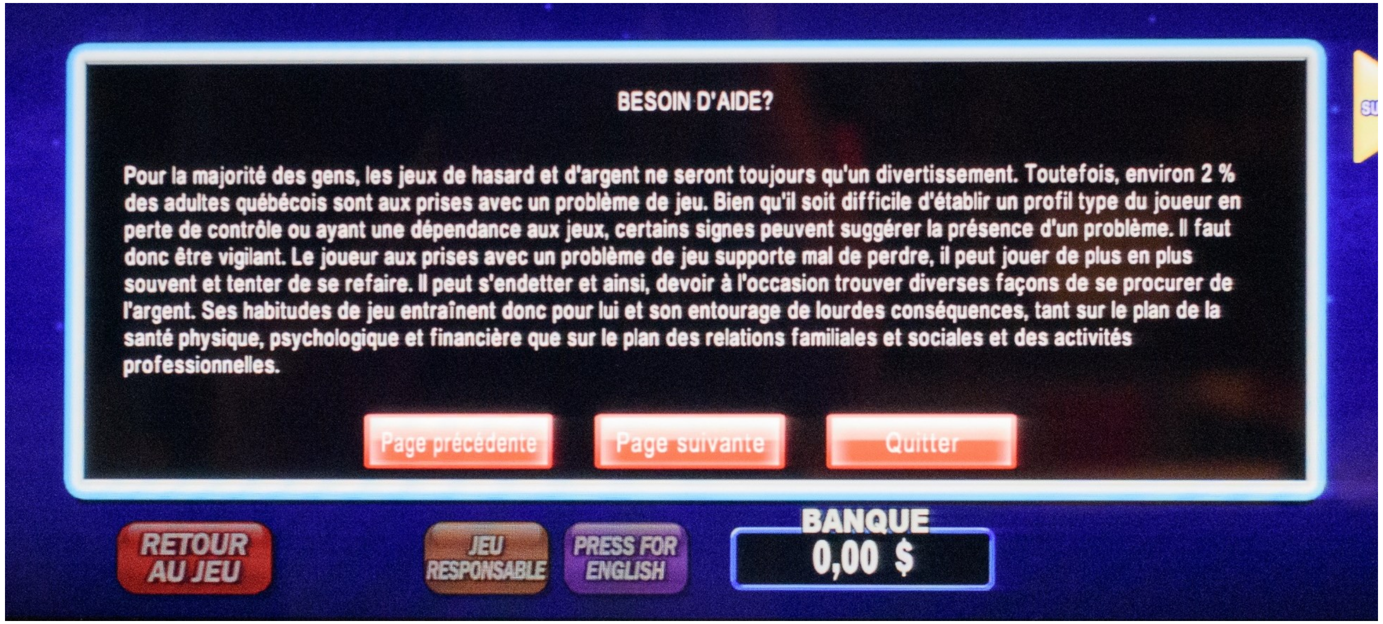


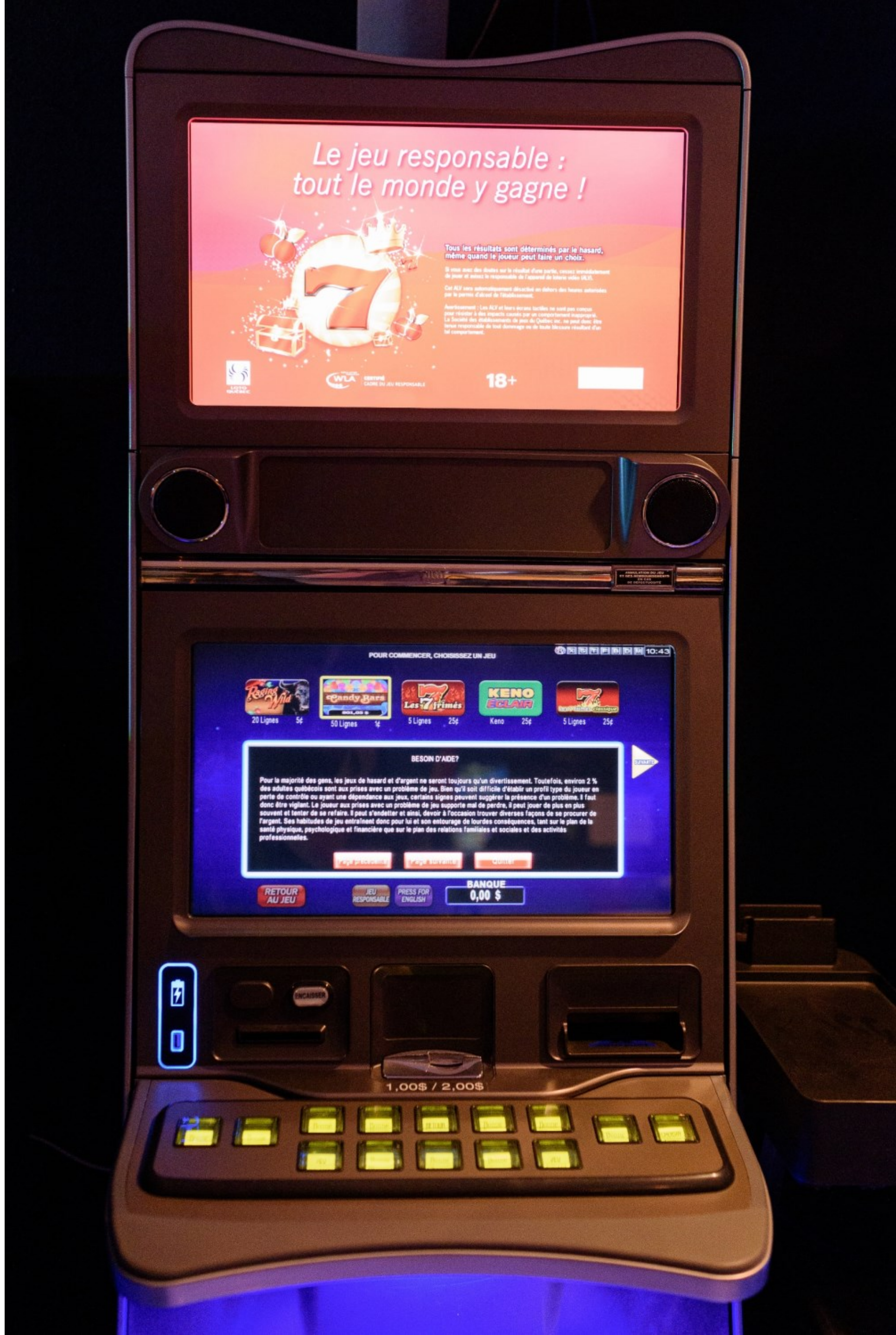

# Responsible gambling information – Overcoming social isolation

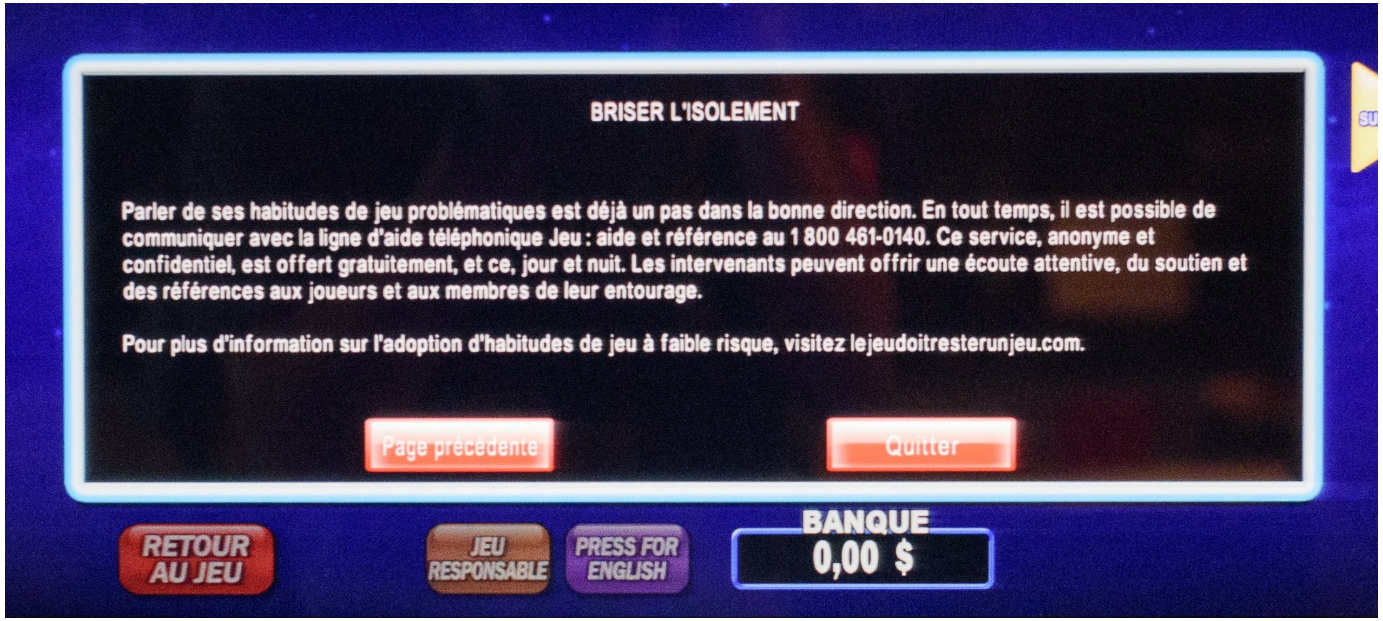


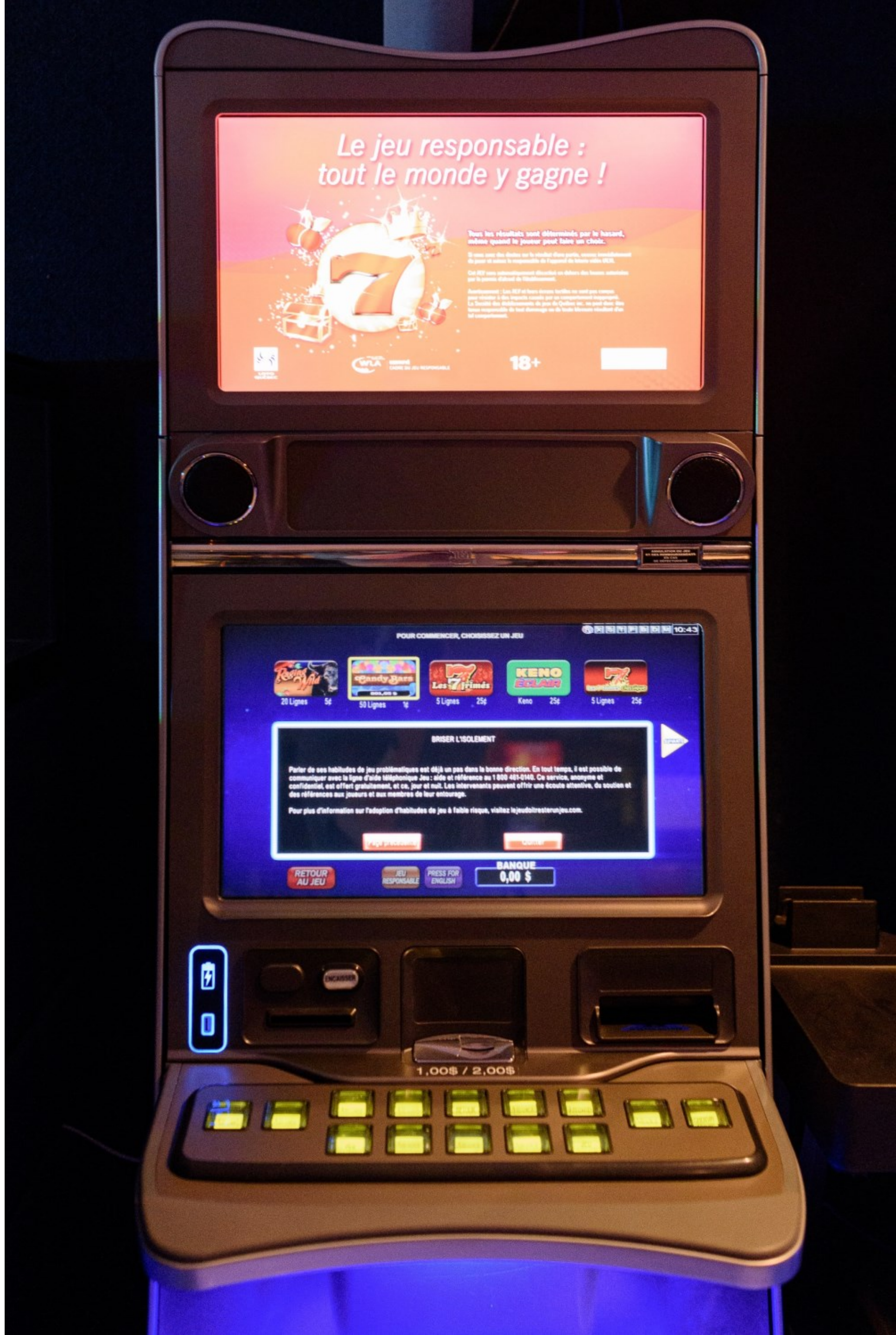

Supplement: Multimedia Appendix 2 [file resprot-v14-e75068-s002.pdf]
