# Supplementary material for: Effects of Prevention Messages for Electronic Gambling Machines on Behaviors and Cognitions: Protocol for a Two-Arm Stratified Block: Randomized Controlled Study
Source: JMIR Res Protoc. 2025 Nov 10;14:e75068. doi: 10.2196/75068 (PMC12599998; doi:10.2196/75068)

Prevention pop-up message – Template

Warning heading (translate from french to "caution")

Warning heading pictogram

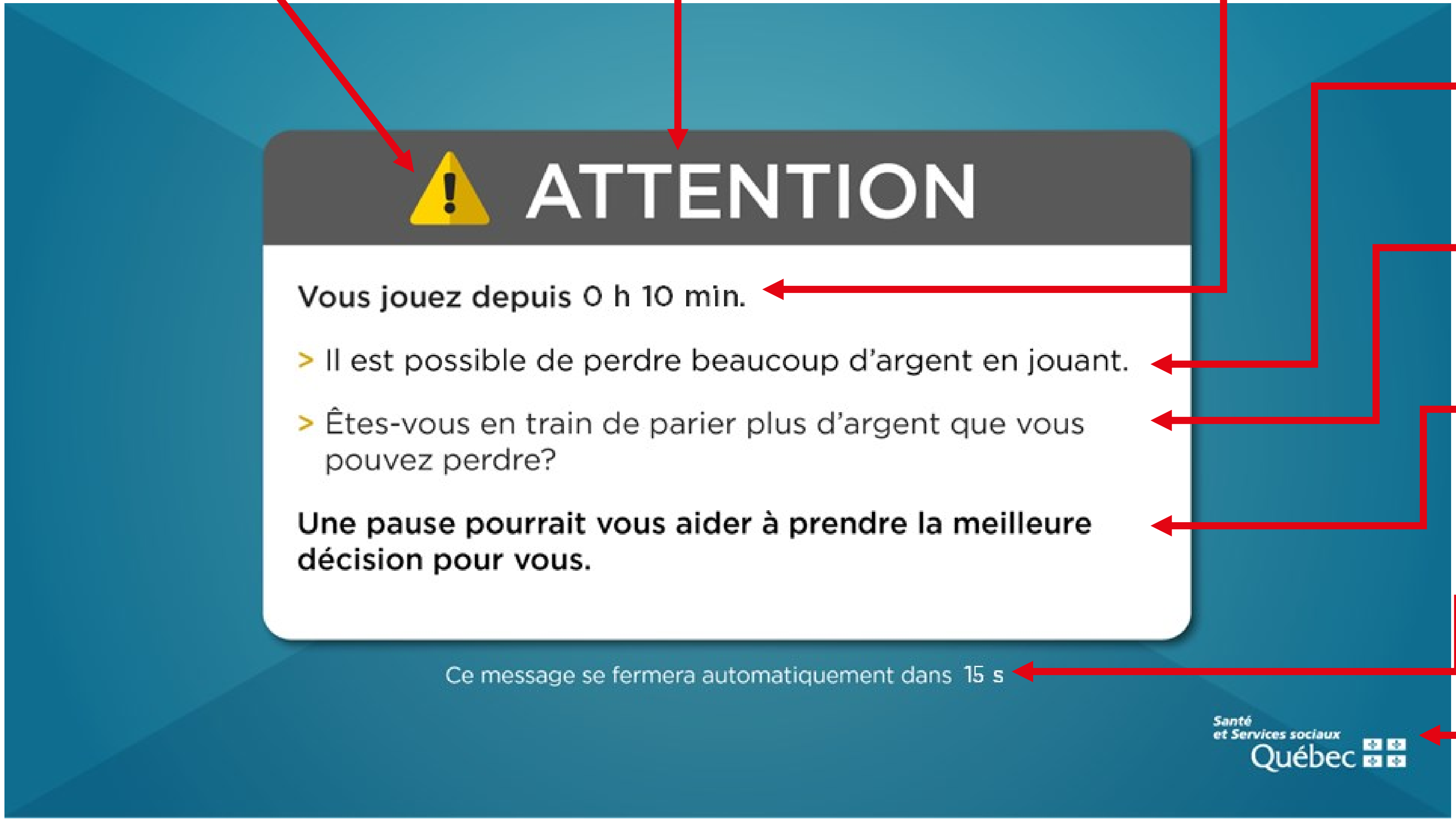

Time spent gambling since the beginning of the session

Information (never changes): "Gambling could lead to serious monetary loss"

Self-appraisal question: 4 versions

Advice (never changes): "A break could help you choose what is best for yourself"

Countdown indicating time left before the message closes itself (starts at 15 s)

Local government Health Department logo

Prevention pop-up message – V1

Self-appraisal question: "Are you currently betting more money than you could afford?"

1

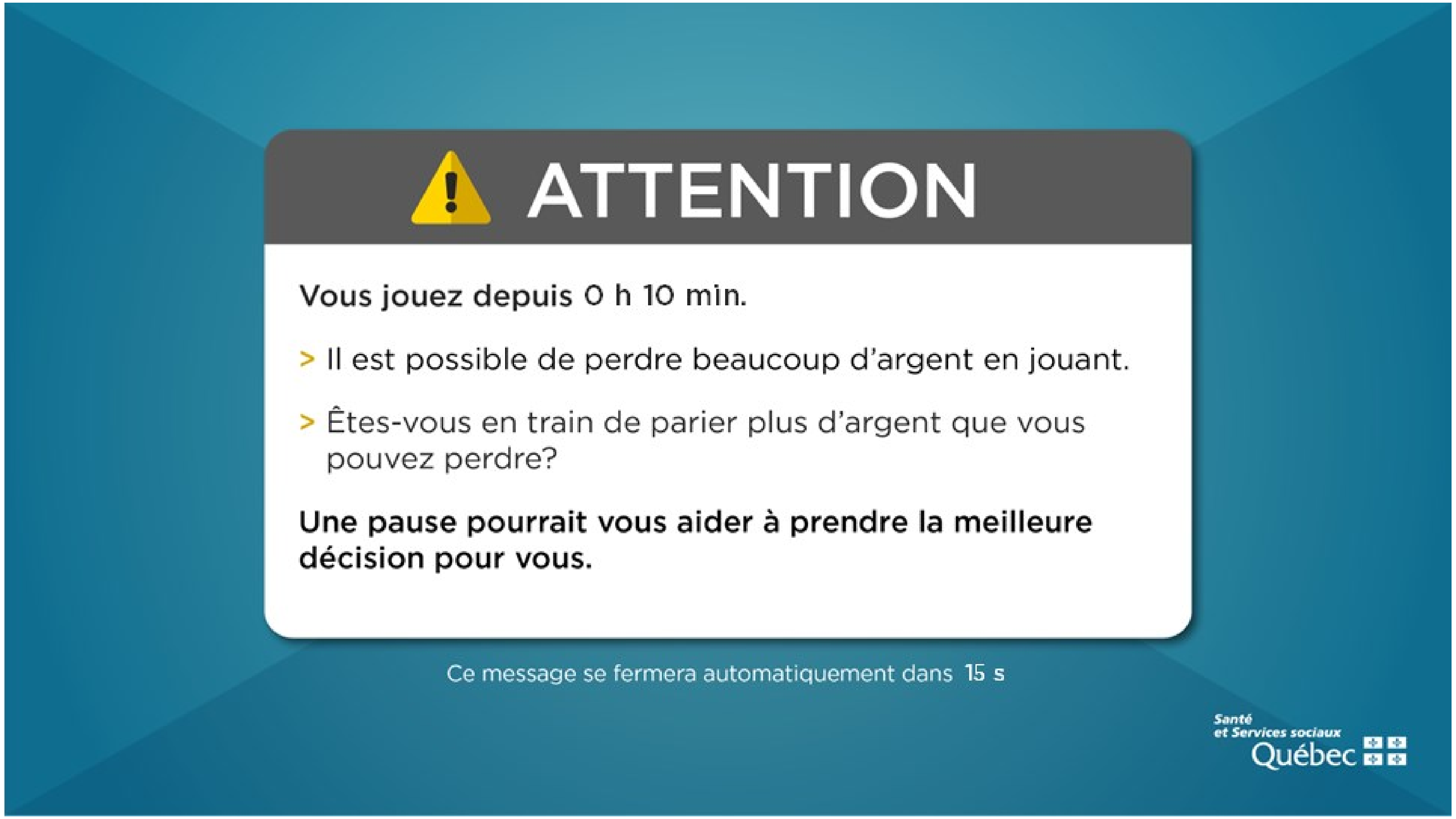

Prevention pop-up message – V2

Self-appraisal question: "Are you currently betting more money than planned?"

2

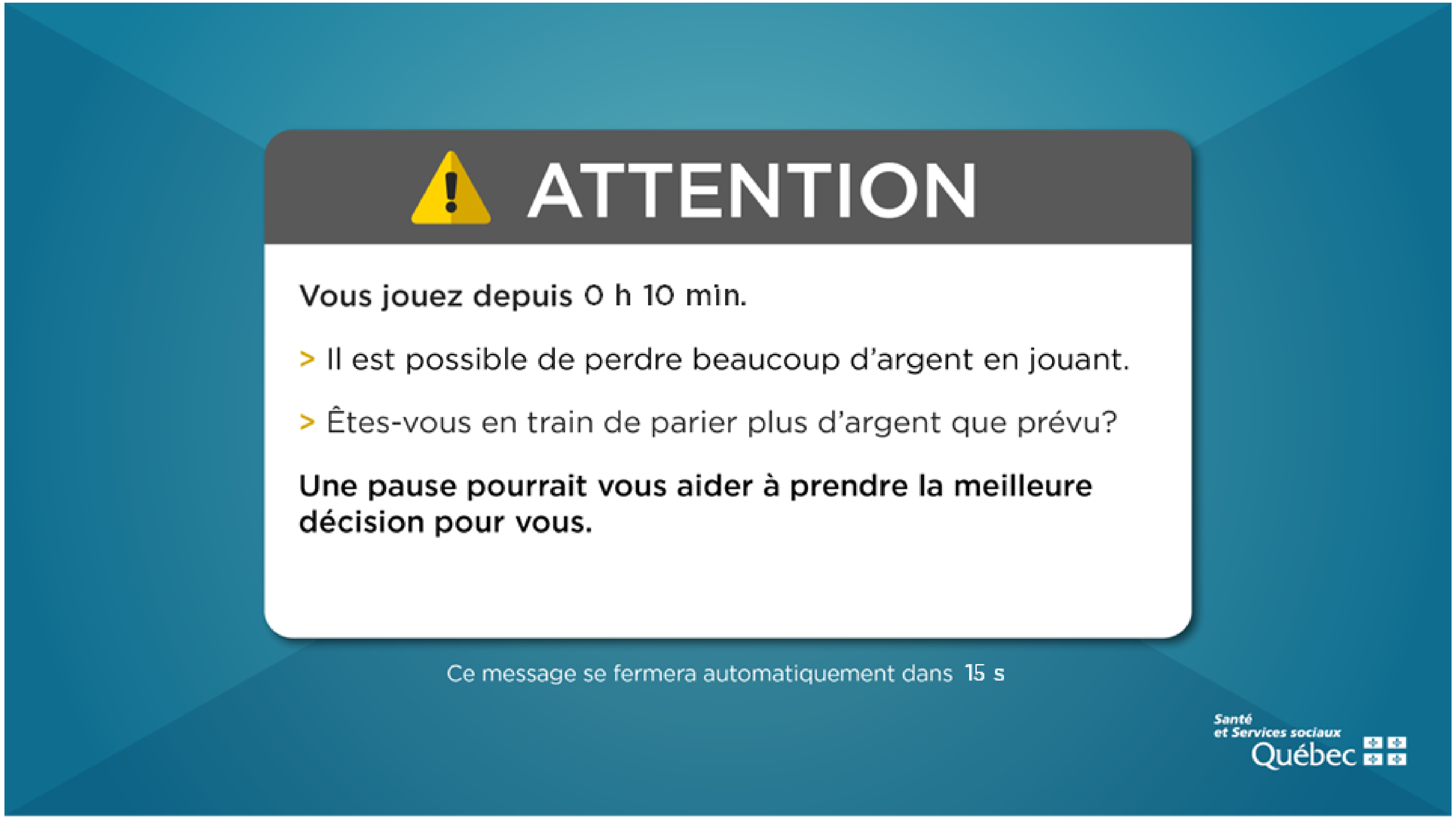

Prevention pop-up message – V3

Self-appraisal question: "Is it currently a good idea to keep playing?"

3

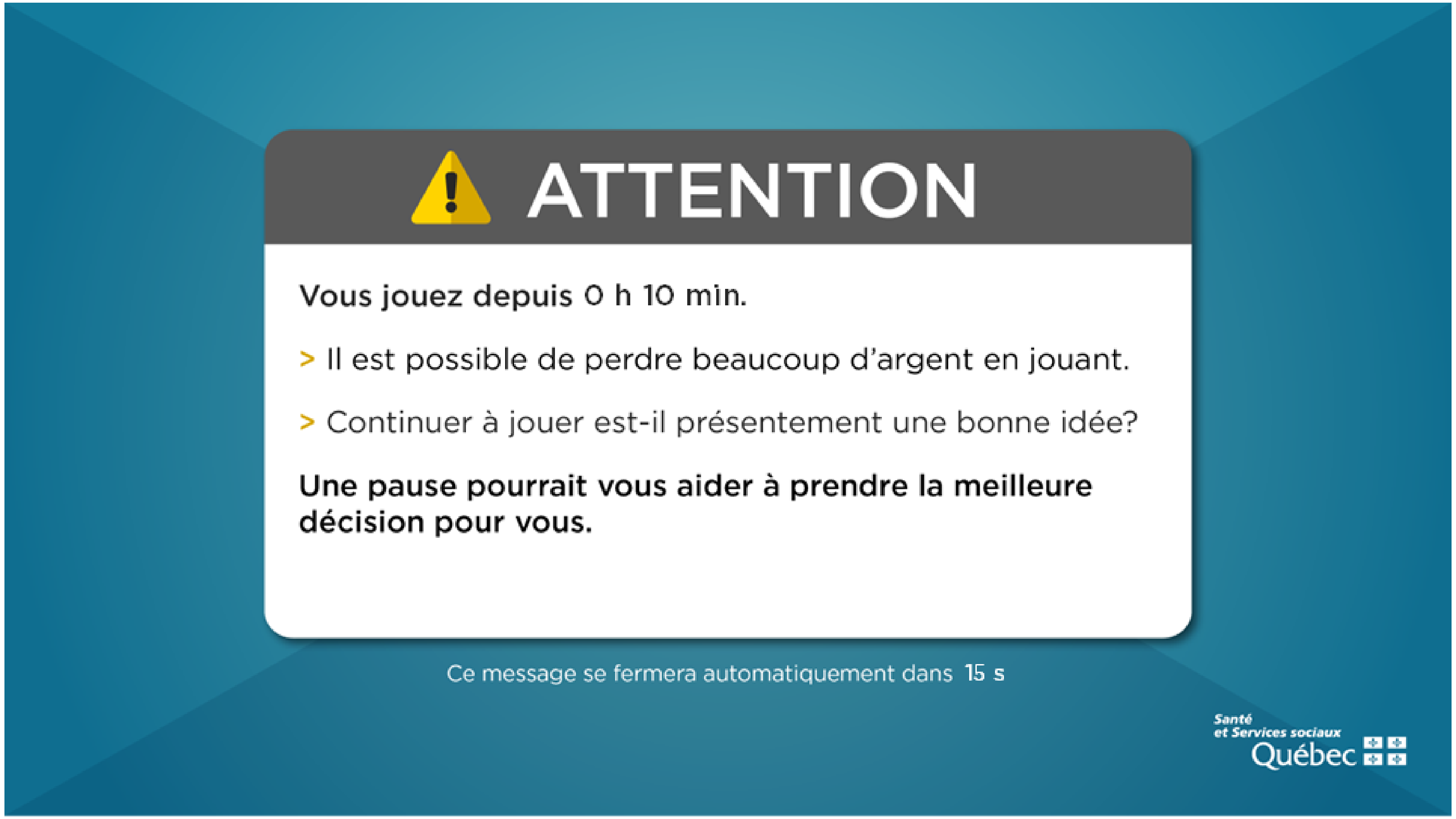

Prevention pop-up message – V4

Self-appraisal question: "Are you currently feeling in control of your decisions?"

4

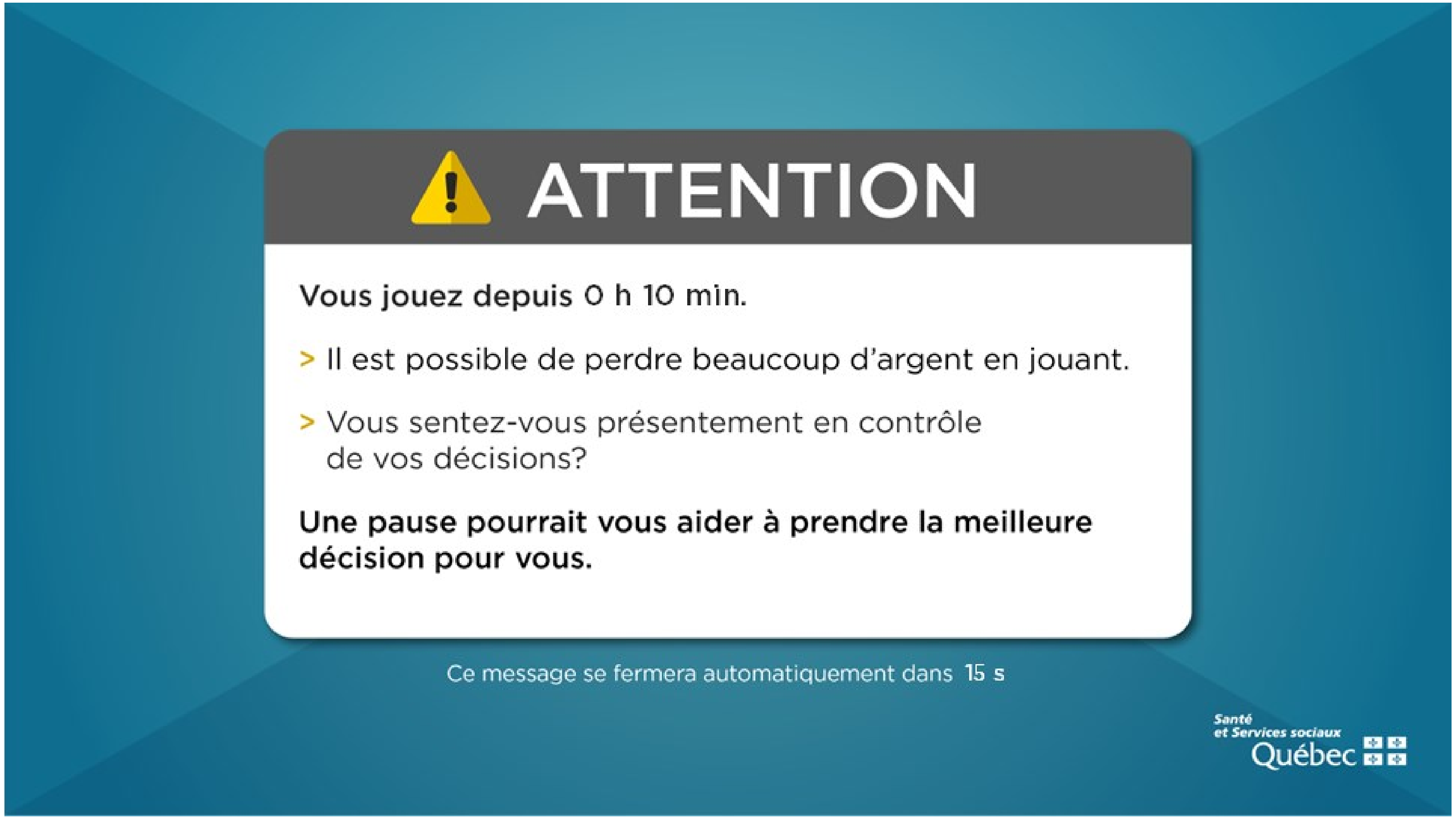

Supplement: Multimedia Appendix 3 [file resprot-v14-e75068-s003.pdf]
